# Cortical and subcortical mapping of the human allostatic–interoceptive system using 7 Tesla fMRI

**DOI:** 10.1038/s41593-025-02087-x

**Published:** 2025-10-23

**Authors:** Jiahe Zhang, Danlei Chen, Philip Deming, Tara Srirangarajan, Jordan E. Theriault, Philip A. Kragel, Ludger Hartley, Kent M. Lee, Kieran McVeigh, Tor D. Wager, Lawrence L. Wald, Ajay B. Satpute, Karen S. Quigley, Susan Whitfield-Gabrieli, Lisa Feldman Barrett, Marta Bianciardi

**Affiliations:** 1 Department of Psychology, Northeastern University, Boston, MA USA; 2 Department of Psychiatry, Massachusetts General Hospital, Boston, MA USA; 3 Department of Psychology, Stanford University, Stanford, CA USA; 4 Department of Biology, Northeastern University, Boston, MA USA; 5 Department of Radiology, Athinoula A. Martinos Center for Biomedical Imaging, Massachusetts General Hospital, Boston, MA USA; 6 Department of Psychology, Emory University, Atlanta, GA USA; 7 Department of Psychological and Brain Sciences, Dartmouth College, Hanover, NH USA; 8 Division of Sleep Medicine, Harvard University, Boston, MA USA

**Keywords:** Neural circuits, Psychology

## Abstract

The brain continuously anticipates the body’s energetic needs and prepares to meet them before they arise—a process called allostasis. To support allostasis, the brain continually models the body’s sensory state, a process known as interoception. Here we replicate and extend a large-scale system that supports allostasis and interoception in the human brain using ultrahigh precision 7 Tesla functional magnetic resonance imaging (*n* = 90), improving precision in subgenual and pregenual anterior cingulate topography and expanding brainstem nuclei mapping. Our functional connectivity analyses provide corroborating evidence for more than 96% of the anatomical connections documented in nonhuman animal tract-tracing studies. This system also includes regions of dense intrinsic connectivity throughout the system, some of which were identified previously as part of the backbone of neural communication across the brain. These results reinforce the existing evidence for a whole-brain system that supports the modeling and regulation of the body’s internal milieu.

## Abstract (web summary)

The brain is constantly monitoring the systems in the body. Here the authors use 7 Tesla functional magnetic resonance imaging to map a large-scale brain system for body regulation in humans, including brainstem nuclei, and confirm many monosynaptic connections traced in nonhuman animals.

## Main

The brain efficiently regulates and coordinates the systems of the body as it continually interfaces with an ever-changing and only partly predictable world. Various lines of research, including tract-tracing studies of nonhuman animals^1,2^, discussions of predictive processing^3–6^ and research on the central control of autonomic nervous system function^7–11^, all suggest the existence of a unified, distributed brain system that anticipates the metabolic needs of the body and prepares to meet those needs before they arise, a process called allostasis^12^ (for recent reviews, see refs. ^13,14^). Allostasis is not a condition or a state of the body, but rather the process by which the brain efficiently coordinates and regulates the various systems of the body^12^. Just as somatosensory and other exteroceptive sensory signals are processed in the service of skeletomotor control, the brain is thought to model the internal sensory conditions of the body (that is, the internal milieu) in the service of allostasis, a process known as interoception^15–18^.

**Fig. 1:**
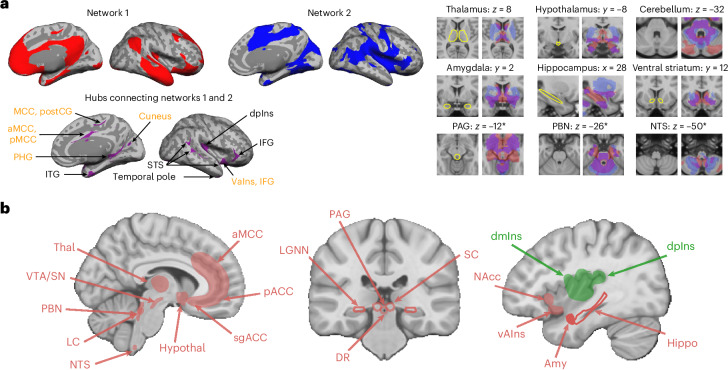
Key cortical and subcortical regions involved in interoception and allostasis. **a**, Using 3 Tesla fMRI resting state connectivity, we showed a unified system consisting of the default mode network (in red) and salience network (in blue), which overlapped in many key cortical visceromotor allostatic regions (in purple) that also serve as ‘rich club’ hubs (labeled in yellow), in addition to a portion of primary interoceptive cortex (dpIns; left)^19^. We reported the system’s connectivity to some subcortical regions known to have a role in control of the autonomic nervous system, the immune system and the endocrine system, such as the thalamus, hypothalamus, hippocampus, ventral striatum, PAG, PBN and NTS (for example, refs. ^20–26^; right)^19^. * denotes brainstem regions. Panel **a** is reproduced with permission from ref. ^19^. **b**, Expanded set of seed regions used in the present analysis. Hippo, hippocampus; hypothal, hypothalamus; IFG, inferior frontal gyrus; ITG, inferior temporal gyrus; PHG, parahippocampal gyrus; postCG, postcentral gyrus; STS, superior temporal sulcus; Thal, thalamus.

Using resting state functional magnetic resonance imaging (fMRI) in three samples totaling almost 700 human participants scanned at 3 Tesla^19^, we previously identified a distributed allostatic–interoceptive system consisting of two well-known intrinsic networks, the default mode and salience networks, overlapping in many key cortical visceromotor allostatic regions that also serve as ‘rich club’ hubs that have been implicated as the ‘backbone’ for neural communication throughout the brain (Fig. 1a). Our investigation was guided by the anatomical tracts identified in published studies of macaques and other nonhuman mammals (Table 2 in ref. ^19^). This study was more cortically focused, examining the functional connectivity of primary interoceptive cortex spanning the dorsal mid insula (dmIns) and dorsal posterior insula (dpIns), as well as key allostatic regions in the cerebral cortex that are directly connected to the brainstem regions that are known to be responsible for controlling the motor changes in the viscera (that is, visceromotor cortical regions), such as the anterior midcingulate cortex (aMCC), pregenual anterior cingulate cortex (pACC), subgenual ACC (sgACC) and agranular insular cortex (also known as ventral anterior insula (vAIns), which is also posterior orbitofrontal cortex), as well as the dorsal amygdala (dAmy) containing the intercalated bodies and the central nucleus (Fig. 1a). Our 3 Tesla analysis yielded a replicable, integrated system consisting of two well-known intrinsic networks, in addition to primary interoceptive cortex. We did explore some aspects of the system’s subcortical extent, including the thalamus, hypothalamus, hippocampus, ventral striatum, periaqueductal gray (PAG), parabrachial nucleus (PBN) and nucleus tractus solitarius (NTS), all regions known to have a role in control of the autonomic nervous system, the immune system and the endocrine system (for example, refs. ^20–26^), but our ability to more extensively map the midbrain and brainstem extents of the system was limited by the use of 3 Tesla imaging.

In the present study, we replicated and extended evidence for the allostatic–interoceptive system (Fig. 1b) using ultrahigh field (7 Tesla) MRI, which allows data acquisition with higher spatial resolution (1.1-mm isotropic), better signal-to-noise ratio (SNR^27–29^) and increased sensitivity in mapping functional connectivity of brainstem nuclei involved in arousal, motor and other vital processes (for example, autonomic, nociceptive and sensory^30,31^). This is particularly important given the increasing importance of the allostatic–interoceptive system as a tool for investigating interoception and allostasis in basic brain function, both in neurotypical samples and in specific populations (for example, refs. ^32–35^). In addition, the research indicates that regions in this system are also important for a wide range of psychological domains, including cognition, emotion, pain, decision-making and perception (Fig. 5 in ref. ^19^; see also refs. ^36–38^), suggesting the hypothesis that allostatic and interoceptive signals may have a more fundamental role in shaping basic brain dynamics (for discussion, see refs. ^5,39–41^).

Thus, we tested within-system functional connectivity in 90 human participants (age range = 18–40 years, mean = 26.9 years, s.d. = 6.2 years; 40 females) using a fast low-angle excitation echo-planar technique sequence shown to reduce artifacts and improve temporal SNR^24,42^. This approach allowed a more precise mapping of connectivity for regions with known signal issues at 3 Tesla, such as the sgACC (low SNR), amygdala (noise from adjacent veins^43^), columns within the PAG (noise from adjacent aqueduct) and other small structures that could be particularly influenced by partial volume effects. We took advantage of the recently developed, much improved and validated in vivo brainstem and diencephalic nuclei atlases^44–48^ to guide our analysis. This was crucial because our hypotheses were specifically derived from published tract-tracing studies of macaques and other nonhuman mammals that establish structural pathways carrying ascending interoceptive signals from the periphery, for example, through the vagus nerve, to subcortical and cortical regions of the allostatic–interoceptive system (Supplementary Table 1). Extending^19^, we more extensively examined the intrinsic connectivity of subcortical nuclei such as mediodorsal thalamus (mdThal), hypothalamus, dAmy, hippocampus, ventral striatum, PAG, PBN and NTS (in the medullary viscero-sensory-motor (VSM) nuclei complex, which also includes the dorsal motor nucleus of the vagus, nucleus ambiguus and hypoglossal nucleus), in addition to considering the connectivity of dorsal raphe (DR), substantia nigra (SN), ventral tegmental area (VTA), locus coeruleus (LC), superior colliculus (SC) and lateral geniculate nucleus (LGN). The DR, SN, VTA and LC are midbrain and pontine monoamine-producing nuclei that contribute to relaying the body’s metabolic status to the cortex^49^. The SC and LGN are not traditionally considered to be directly involved in interoception and allostasis, but they share anatomical connections with key visceromotor regulation regions in the system (Supplementary Table 1; refs. ^50–55^). For example, neurons in the intermediate and deep layers of the SC are connected to aMCC^56^, hypothalamus^57,58^ and PAG^59^, and have been directly implicated in skeletomotor^60,61^ and visceromotor^62,63^ actions that facilitate approach or avoidance behaviors. The SC is also thought to be a major point of sensory-motor integration and is associated with affective feelings^64,65^. The LGN receives interoceptive inputs from the PAG^52^ and PBN^55,66,67^, and shares monosynaptic connections with the hypothalamus^68^ and pACC^69^. We also examined connectivity patterns for subregions of the PAG, hippocampus, SC and hypothalamus rather than as a single region of interest (ROI) as discussed in ref. ^19^ given their functional heterogeneity^70,71^ and differential involvement in allostasis (for example, refs. ^72–74^).

## Results

We thus used a bootstrapping strategy to identify weak yet reliable signals that are important when examining cortical–subcortical connections in brain-wide analyses. For each of the 1,000 iterations, we randomly resampled 80% of the participants (*n* = 72) and identified, for each seed region, blood oxygen level dependent (BOLD) signal correlations for all voxels in the brain that survived a voxel-wise threshold of *P* < 0.05. We calculated discovery maps for each seed region that included both cortical and subcortical connections. We also calculated the similarities in the spatial topography among all the maps and subjected each resulting similarity matrix to *k-*means clustering analysis to characterize the allostatic–interoceptive network. We expected stronger connectivity among cortical seeds compared to subcortical seeds due to the latter’s noisier time courses and potential partial volume effects, which would result in lower correlations for smaller regions.

### Cortico-cortical intrinsic connectivity

**Fig. 2:**
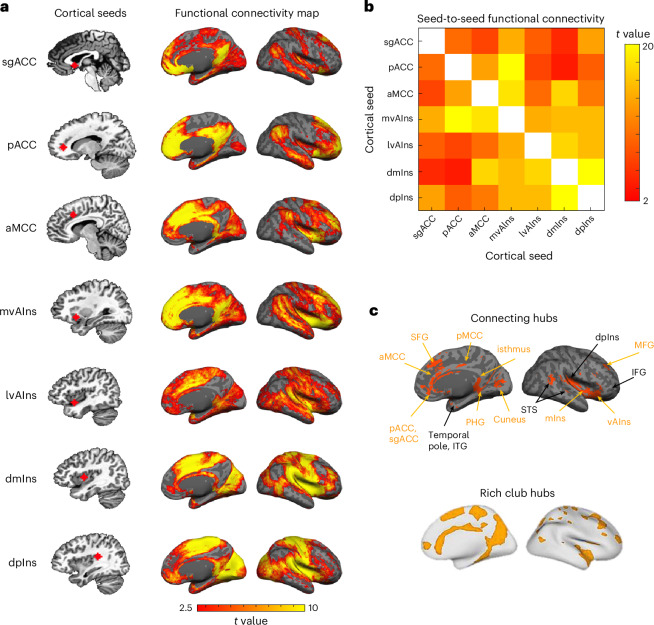
Cortico-cortical functional connectivity within the allostatic–interoceptive system. **a**, Left column shows cortical seed locations and right column shows group-level *t-*value maps (*n* = 90) masked by voxels that showed positive connectivity (two-tailed *t* test, *P* < 0.05) with the seed in more than 950 iterations (of 1,000) by resampling 80% of the sample in each iteration (*n* = 72). **b**, Seed-to-seed functional connectivity matrix shows connectivity strength between each pair of the cortical seeds (two-tailed *t* test, *P* < 0.05, uncorrected; white color indicates correlation = 1; *n* = 90). **c**, The allostatic–interoceptive system showed connecting regions in all the a priori interoceptive and visceromotor control regions. Connecting regions belonging to the ‘rich club’ are labeled in yellow. ‘Rich club’ hubs image in panel **c** is adapted with permission from ref. ^105^. lvAIns, lateral vAIns; mIns, mid insula; mvAIns, medial vAIns.

**Extended Data Fig. 1:**
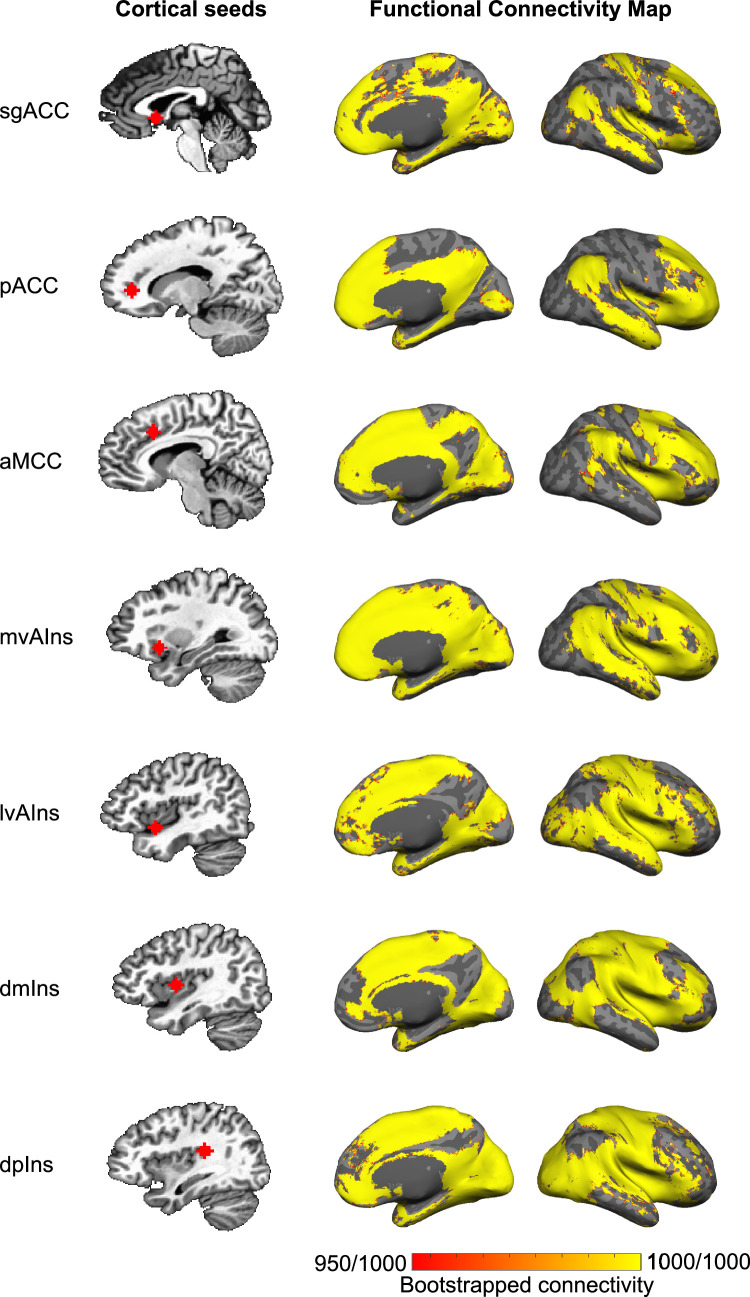
Bootstrapped cortico-cortical functional connectivity maps. Bootstrapped functional connectivity maps depict all voxels whose time course was correlated (two-tailed t-test, *p* < 0.05) with that of the seed in more than 950 iterations (out of 1000) by resampling 80% of the sample in each iteration (*n* = 72). aMCC: anterior midcingulate cortex; dmIns: dorsal mid insula; dpIns: dorsal posterior insula; lvAIns: lateral ventral anterior insula; mvAIns: medial ventral anterior insula; pACC: pregenual anterior cingulate cortex; sgACC: subgenual anterior cingulate cortex.

We first examined the hypothesized functional connectivity according to the published anatomical connections. As expected, we successfully replicated all of the cortico-cortical connections we previously observed with 3 Tesla imaging (Fig. 2 and Supplementary Table 1)^19^. In addition, we observed reciprocal intrinsic connectivity (that is, connectivity map of one region includes a cluster in the other region and vice versa) between the lateral vAIns and pACC, between the sgACC and aMCC, and between the dmIns and portions of cingulate cortex (sgACC and pACC; Fig. 2a and see Extended Data Fig. 1 for bootstrapped maps identifying connectivity surviving *P* value of <0.05 in more than 95% of subsampled analyses), extending the allostatic–interoceptive system to include more of the anatomical connections documented in tract-tracing studies in nonhuman mammals^75–78^. All of these observations were confirmed by seed-to-seed connectivity strength calculation (Fig. 2b). Cortico-cortical functional connectivity within the allostatic–interoceptive system, as evidenced by the cortical maps and seed-to-seed connectivity matrix based on our sample, confirmed 100% of the monosynaptic connections documented in published tract-tracing studies of nonhuman animals (see Supplementary Table 1 and references therein).

**Fig. 3:**
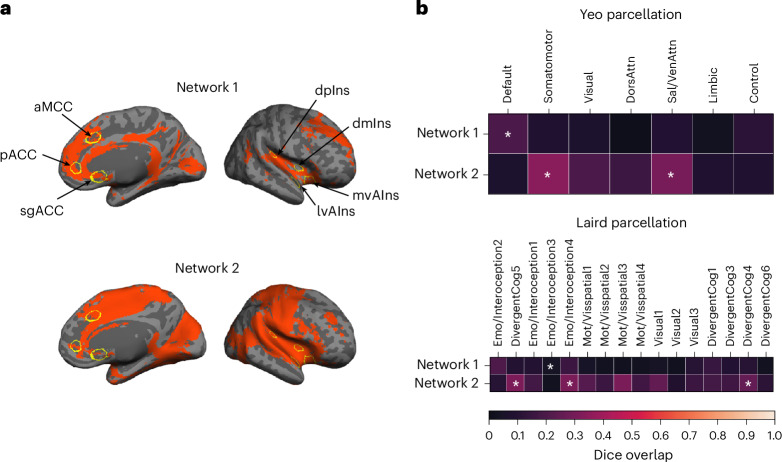
The two large-scale intrinsic networks composing the cortical allostatic–interoceptive system correspond to the default mode network and salience/somatomotor networks. **a**, The cortical allostatic–interoceptive system is composed of two large-scale intrinsic networks. The *k*-means clustering (*k* = 2, 1,000 iterations) yielded the most optimal solution, where Network 1 (resembling the default mode network) included a cluster of maps seeded in the sgACC, aMCC, pACC and mvAIns, and Network 2 (resembling the salience network) included a cluster of maps seeded in the lvAIns, dmIns and dpIns. All displayed maps result from the conjunction of binarized maps (two-tailed *t* test, *P* < 0.05) in the same cluster. Cortical ROIs are outlined in yellow (ROI names are labeled in the top panel). **b**, We computed Dice overlap between network maps and the Yeo 7-network cortical parcellation^149^ using the Network Correspondence Toolbox (https://github.com/rubykong/cbig_network_correspondence)^150^. In the grids, cells with significant Dice overlap at *P* < 0.05 (that is, showing substantial correspondence) are denoted with an asterisk. Network 1 showed significant Dice overlap solely with the default mode network, while Network 2 showed significant Dice overlap with the salience/ventral attention network and somatomotor network.

Next, we binarized the cortical connectivity maps for all cortical seeds (*P* < 0.05) and computed their conjunction to identify the connecting cortical regions (Fig. 2c). A *k*-means clustering analysis (optimal *k* = 2 based on the Calinski–Harabasz criterion^79^) on the cortical maps replicated^19^, such that the system included two subsystems, one corresponding to the default mode network (that is, the dorsomedial prefrontal cortex, posterior cingulate cortex (PCC), and dorsolateral prefrontal cortex) and the other corresponding to the salience (that is, anterior to MCC, anterior insula, supramarginal gyrus, supplementary motor area) and somatomotor networks (that is, precentral gyrus, postcentral gyrus, superior temporal gyrus (STG); see details in Fig. 3); this ensemble of brain regions is sometimes referred to as the cingulo-opercular network or the action-mode network^80^. This procedure also enabled us to identify any regions that could be reliably included in the intrinsic connectivity of the system. We replicated all the connecting ‘hub’ regions reported at 3 Tesla discussed in ref. ^19^ (that is, portions of aMCC/pMCC, inferior frontal gyrus (IFG), vAIns, dpIns, temporal pole, inferior temporal gyrus, superior temporal sulcus, parahippocampal gyrus (PHG) and cuneus) with the exception of medial postcentral gyrus. We also newly identified the entire ACC (including subgenual and pregenual extents), PCC, a greater extent of the insula (including mid insula), as well as some portions of medial superior frontal gyrus (SFG) and middle frontal gyrus (MFG) as allostatic–interoceptive system ‘hubs’. A majority of the allostatic–interoceptive system’s connecting hubs have been identified as members of the ‘rich club’ in the connectomics literature, defined as high-degree nodes showing denser interconnections among themselves than are lower-degree nodes^81^. The rich club hubs have a key role in global information integration across the brain and therefore may serve as the backbone for global communication within the brain^82^, suggesting that allostatic and interoceptive processes may be at the core of the brain’s computational architecture.

### Subcortico-cortical intrinsic connectivity

**Fig. 4:**
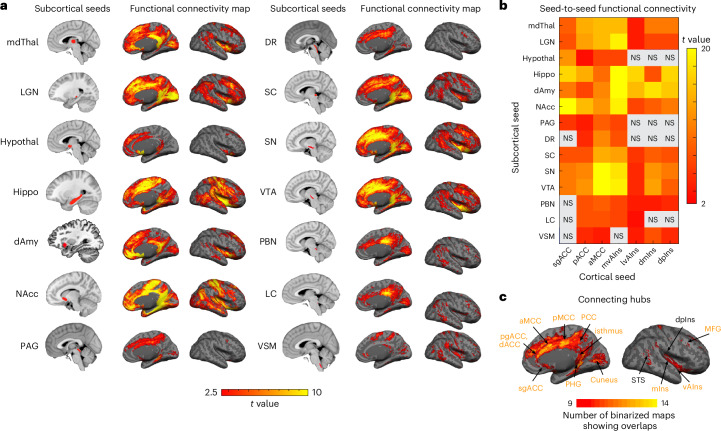
Subcortico-cortical intrinsic connectivity within the allostatic–interoceptive system. **a**, Left column shows subcortical seed locations and right column shows group-level *t-*value maps (*n* = 90) masked by voxels that showed positive connectivity (two-tailed *t* test, *P* < 0.05) with the seed in more than 950 iterations (of 1,000 iterations) by resampling 80% of the sample in each iteration (*n* = 72). **b**, Seed-to-seed functional connectivity matrix shows connectivity strength between pairs of subcortical and cortical seeds (two-tailed *t* test, *P* < 0.05, uncorrected; gray color indicates subthreshold correlations; *n* = 90). **c**, Conjunction map shows the number of binarized maps (two-tailed *t* test, *P* < 0.05) with shared connecting regions (ranging from 9 to 14). dACC, dorsal ACC.

**Extended Data Fig. 2:**
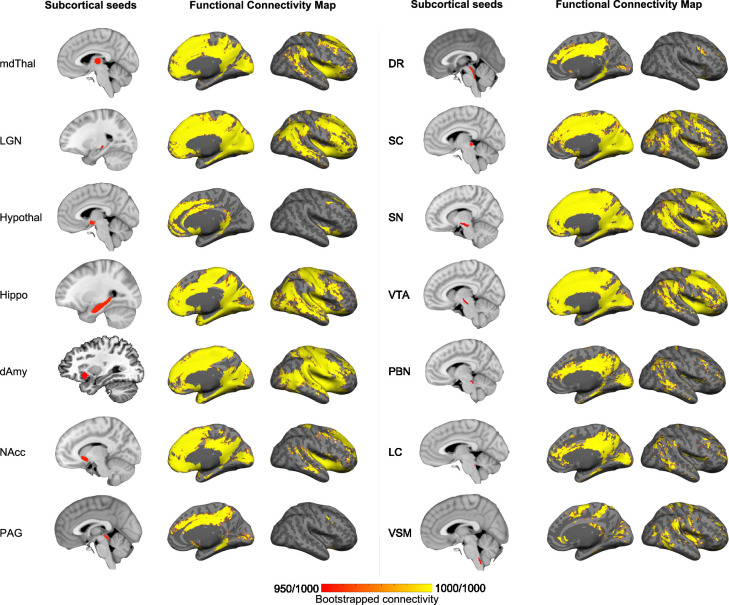
Bootstrapped subcortico-cortical functional connectivity maps. Bootstrapped functional connectivity maps depict all voxels whose time course was correlated (two-tailed t-test, *p* < 0.05) with that of the seed in more than 950 iterations (out of 1000) by resampling 80% of the sample in each iteration (*n* = 72). dAmy: dorsal amygdala; DR: dorsal raphe; hippo: hippocampus; hypothal: hypothalamus; LC: locus coeruleus; LGN: lateral geniculate nucleus; mdThal: mediodorsal thalamus; NAcc: nucleus accumbens; PAG: periaqueductal gray; PBN: parabrachial nucleus; SC: superior colliculus; SN: substantia nigra; VSM: medullary viscero-sensory-motor nuclei complex, including the nucleus tractus solitarius, dorsal motor nucleus of the vagus, nucleus ambiguus, and hypoglossal nucleus; VTA: ventral tegmental area.

In a new analysis that was enabled by newly delineated subcortical seeds^45,48,83^ and that presented considerable challenges at 3 Tesla^19^ due to its coarser spatial resolution and lower SNR^27^, we assessed subcortico-cortical connectivity by visually inspecting cortical discovery maps of the subcortical seeds to confirm topography (Fig. 4a and see Extended Data Fig. 2 for bootstrapped maps identifying connectivity surviving *P* < 0.05 in more than 95% of subsampled analyses) and calculating seed-to-seed connectivity to quantify strength of connection (Fig. 4b). Subcortico-cortical functional connectivity within the allostatic–interoceptive system, as evidenced by the cortical maps and seed-to-seed connectivity matrix based on our sample, confirmed 96% of the monosynaptic connections documented in published tract-tracing studies of nonhuman animals (see Supplementary Table 1 and references therein). There were two exceptions as follows: we did not observe significant, positive functional connectivity between PAG and dmIns/dpIns, or PBN and sgACC, despite known anatomical connections (Supplementary Table 1; refs. ^84,85^). In some instances, averaged time courses between seeds did not correlate significantly (that is, gray squares in Fig. 4b, for example, DR–sgACC); however, connectivity clusters could still be observed in the maps (for example, sgACC cluster in DR-seeded map). Such discrepancies can result from noisy signals within an ROI or specific subportions of an ROI showing significant connectivity. We tested specificity of the allostatic–interoceptive network using a region of superior parietal lobule not known for visceromotor function^19^. This region only showed consistent functional connectivity to the SC^86^, VSM, the hippocampus and the dAmy (Supplementary Table 2).

**Fig. 5:**
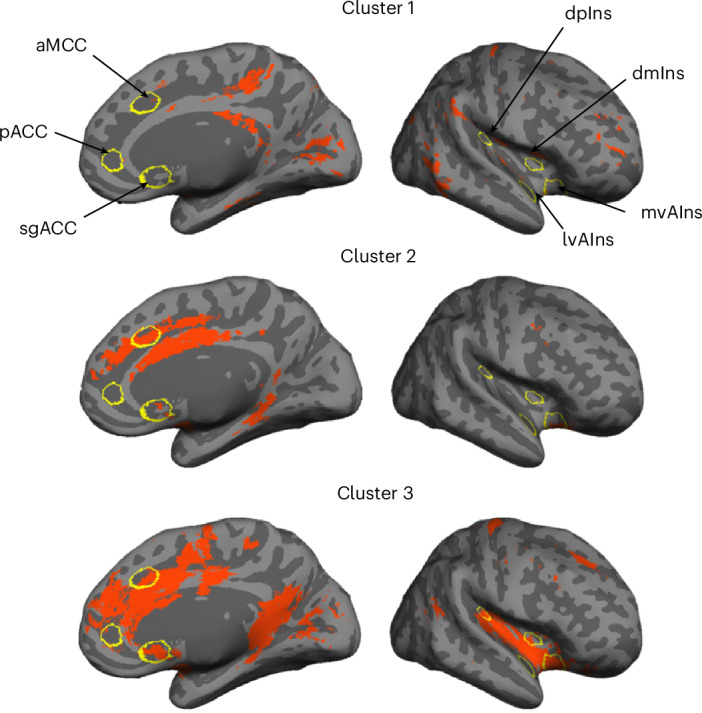
Clustering solution (*k* = 3) for cortical maps of subcortical allostatic–interoceptive seeds. Cluster 1 included maps that were seeded in small lower brainstem ROIs (LC, PBN, VSM). Cluster 2 included maps that were seeded in small upper brainstem ROIs (PAG and DR) and the hypothalamus. Cluster 3 included maps that were seeded in larger subcortical seeds (mdThal, LGN, hippocampus, dAmy, NAcc, SC, SN and VTA). All displayed maps result from the conjunction of binarized maps (*P* < 0.05) in the same cluster. Cortical ROIs are outlined in yellow (ROI names are labeled in the top panel).

As with the cortico-cortical analyses, we conjoined the binarized discovery subcortico-cortical maps (*P* < 0.05) to identify the overlapping cortical connectivity across subcortical seeds (Fig. 4c). Subcortically seeded maps showed connecting regions in hypothesized cingulate and insular regions, as well as in some parts of the MFG and cuneus. We examined a range of *k* values that showed similarly optimal Calinski–Harabasz criterion (*k* = 2 to *k* = 9; Supplementary Note). We retained *k* = 3 for its interpretability. All three clusters included cortical nodes from the default mode and salience networks. Cluster 1 included discovery maps from seeds in the lower brainstem (LC, PBN, VSM), and primarily showed connectivity to the PCC, supramarginal gyrus and some medial and lateral occipital regions (Fig. 5). Cluster 2 included discovery maps from seeds in the upper brainstem (PAG, DR) and the hypothalamus, and showed connectivity to the aMCC and PHG. Cluster 3 included discovery maps from larger seeds in the mdThal, LGN, hippocampus, dAmy, nucleus accumbens (NAcc), SC, SN and VTA, and showed widespread connectivity to the dorsomedial prefrontal cortex, cingulate cortices (sgACC, pgACC, aMCC, isthmus), supplementary motor area, cuneus, insula (anterior, mid and posterior), SFG, central sulcus and angular gyrus.

### Subcortico-subcortical intrinsic connectivity

**Fig. 6:**
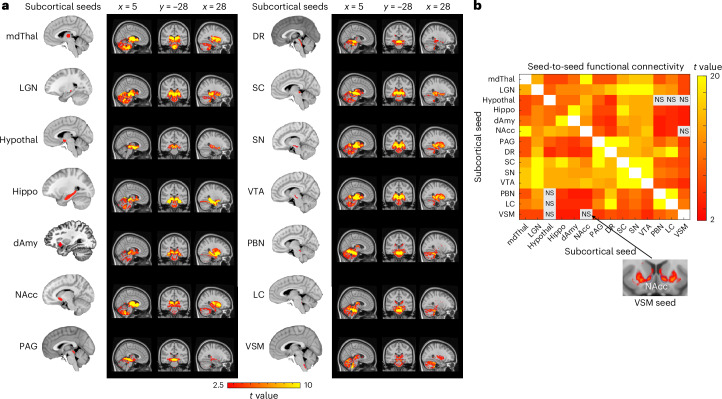
Subcortico-subcortical intrinsic connectivity within the allostatic–interoceptive system. **a**, Left column shows subcortical seed locations and right column shows group-level *t-*value maps (*n* = 90) masked by voxels that showed positive connectivity (two-tailed *t* test, *P* < 0.05) with the seed in more than 950 iterations (of 1,000 iterations) by resampling 80% of the sample in each iteration (*n* = 72). **b**, Seed-to-seed functional connectivity matrix showed connectivity strength between each pair of the subcortical seeds (two-tailed *t* test, *P* < 0.05, uncorrected; white color indicates correlation = 1 and gray color indicates subthreshold correlations; *n* = 90). Several seeds had functional connectivity with a subset of voxels within target ROIs, as shown by binarized maps at *P* < 0.05 (two-tailed *t* test; target ROI outline is shown in blue).

**Extended Data Fig. 3:**
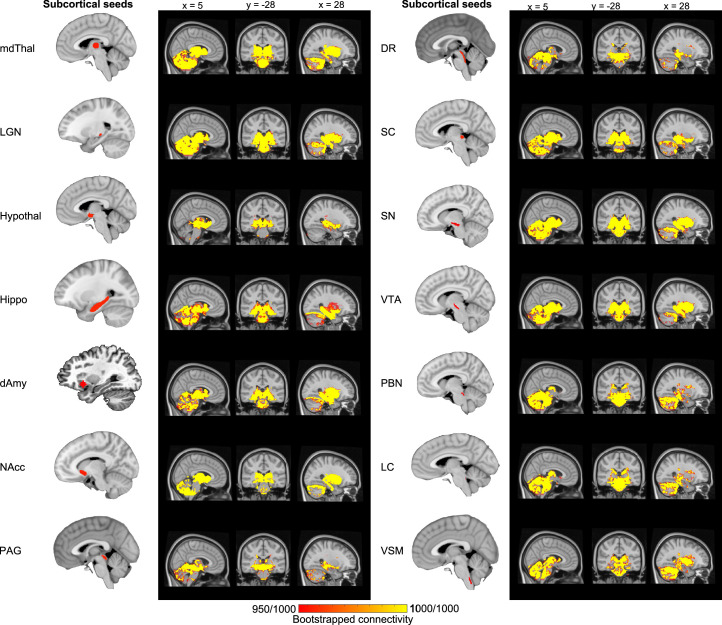
Bootstrapped subcortico-subcortical functional connectivity maps. Bootstrapped functional connectivity depict all voxels whose time course was correlated (two-tailed t-test, *p* < 0.05) with that of the seed in more than 950 iterations (out of 1000) by resampling 80% of the sample in each iteration (*n* = 72). dAmy: dorsal amygdala; DR: dorsal raphe; hippo: hippocampus; hypothal: hypothalamus; LC: locus coeruleus; LGN: lateral geniculate nucleus; mdThal: mediodorsal thalamus; NAcc: nucleus accumbens; PAG: periaqueductal gray; PBN: parabrachial nucleus; SC: superior colliculus; SN: substantia nigra; VSM: medullary viscero-sensory-motor nuclei complex, including the nucleus tractus solitarius, dorsal motor nucleus of the vagus, nucleus ambiguus, and hypoglossal nucleus; VTA: ventral tegmental area.

**Extended Data Fig. 4:**
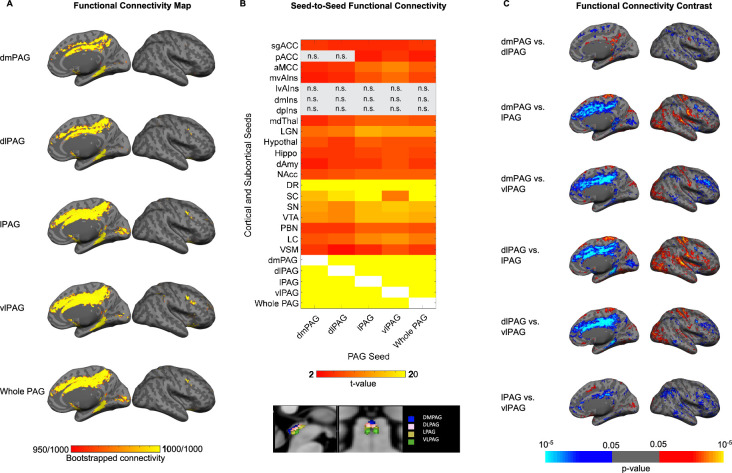
Intrinsic connectivity of PAG and its subregions within the allostatic-interoceptive system. **a**, Bootstrapped connectivity maps obtained from resampling 80% of the sample (*n* = 72) 1000 times. **b**, Connectivity strength between PAG seeds and all other seeds (two-tailed t-test, *p* < 0.05, uncorrected; white color indicates correlation = 1 and gray color indicates subthreshold correlations; *n* = 90). **c**, Contrasts obtained by paired-sample t-tests between subregional maps (*p* < 0.05, uncorrected). lPAG and vlPAG showed more robust and more extensive connectivity than dmPAG and dlPAG, with stronger connectivity especially with aMCC, mvAIns and dAmy. aMCC: anterior midcingulate cortex; dAmy: dorsal amygdala; dmIns: dorsal mid insula; dmPAG: dorsomedial periaqueductal gray; dlPAG: dorsolateral periaqueductal gray; dpIns: dorsal posterior insula; DR: dorsal raphe; hippo: hippocampus; hypothal: hypothalamus; LC: locus coeruleus; LGN: lateral geniculte nucleus; lPAG: lateral periaqueductal gray; lvAIns: lateral ventral anterior insula; mdThal: mediodorsal thalamus; mvAIns: medial ventral anterior insula; NAcc: nucleus accumbens; pACC: pregenual anterior cingulate cortex; PAG: periaqueductal gray; PBN: parabrachial nucleus; SC: superior colliculus; sgACC: subgenual anterior cingulate cortex; SN: substantia nigra; vlPAG: ventrolateral periaqueductal gray; VSM: medullary viscero-sensory-motor nuclei complex, including the nucleus tractus solitarius, dorsal motor nucleus of the vagus, nucleus ambiguus, and hypoglossal nucleus; VTA: ventral tegmental area.

**Extended Data Fig. 5:**
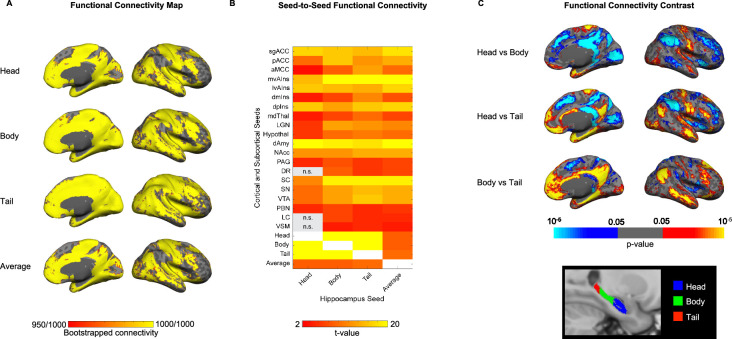
Intrinsic connectivity of the hippocampus and its subregions within the allostatic-interoceptive system. **a**, Bootstrapped connectivity maps obtained from resampling 80% of the sample (*n* = 72) 1000 times. **b**, Seed-to-seed connectivity strength between hippocampal seeds and all other seeds (two-tailed t-test, *p* < 0.05, uncorrected; white color indicates correlation = 1 and gray color indicates subthreshold correlations; *n* = 90). **c**, Contrasts obtained by paired-sample t-tests between subregional maps (two-tailed t-test, *p* < 0.05, uncorrected). Hippocampal head and body showed stronger connectivity to default mode nodes such as the MPFC, PCC, AG and lateral temporal cortex. Hippocampal body and tail showed stronger connectivity to salience nodes such as ACC, PCC, SMA, MFG and SMG. ACC: anterior cingulate cortex; aMCC: anterior midcingulate cortex; dAmy: dorsal amygdala; dmIns: dorsal mid insula; dpIns: dorsal posterior insula; DR: dorsal raphe; hypothal: hypothalamus; LC: locus coeruleus; LGN: lateral geniculate nucleus; lvAIns: lateral ventral anterior insula; mdThal: mediodorsal thalamus; MFG: middle frontal gyrus; mvAIns: medial ventral anterior insula; NAcc: nucleus accumbens; pACC: pregenual anterior cingulate cortex; PAG: periaqueductal gray; PBN: parabrachial nucleus; PCC: posterior cingulate cortex; SC: superior colliculus; sgACC: subgenual anterior cingulate cortex; SMA: supplementary motor area; SMG: supramarginal gyrus; SN: substantia nigra; VSM: medullary viscero-sensory-motor nuclei complex, including the nucleus tractus solitarius, dorsal motor nucleus of the vagus, nucleus ambiguus, and hypoglossal nucleus; VTA: ventral tegmental area.

**Extended Data Fig. 6:**
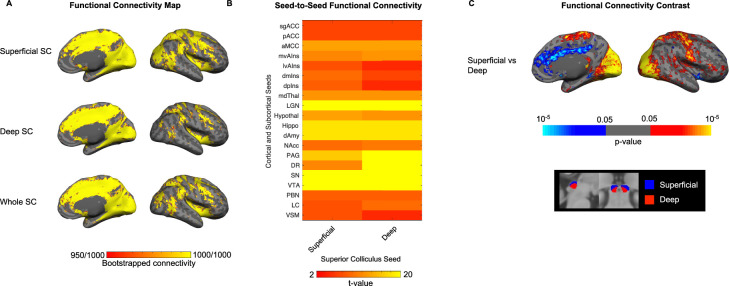
Intrinsic connectivity of the superficial and deep layers of the SC within the allostatic-interoceptive system. **a**, Bootstrapped connectivity maps obtained from resampling 80% of the sample (*n* = 72) 1000 times. **b**, Seed-to-seed connectivity strength between SC subregions and all other seeds (two-tailed t-test, *p* < 0.05, uncorrected; *n* = 90). **c**, Contrasts obtained by paired-sample t-tests between subregional maps (two-tailed t-test, *p* < 0.05, uncorrected). Superficial SC showed stronger connectivity to primary sensory cortices in the posterior insular, occipital and pericentral regions. Deep SC showed stronger connectivity to allostatic-interoceptive hubs in the vAIns and the entire cingulate cortex. aMCC: anterior midcingulate cortex; dAmy: dorsal amygdala; dmIns: dorsal mid insula; dpIns: dorsal posterior insula; DR: dorsal raphe; hippo: hippocampus; hypothal: hypothalamus; LC: locus coeruleus; LGN: lateral geniculate nucleus; lvAIns: lateral ventral anterior insula; mdThal: mediodorsal thalamus; mvAIns: medial ventral anterior insula; NAcc: nucleus accumbens; pACC: pregenual anterior cingulate cortex; PAG: periaqueductal gray; PBN: parabrachial nucleus; PCC: posterior cingulate cortex; SC: superior colliculus; sgACC: subgenual anterior cingulate cortex; SN: substantia nigra; VSM: medullary viscero-sensory-motor nuclei complex, including the nucleus tractus solitarius, dorsal motor nucleus of the vagus, nucleus ambiguus, and hypoglossal nucleus; VTA: ventral tegmental area.

**Extended Data Fig. 7:**
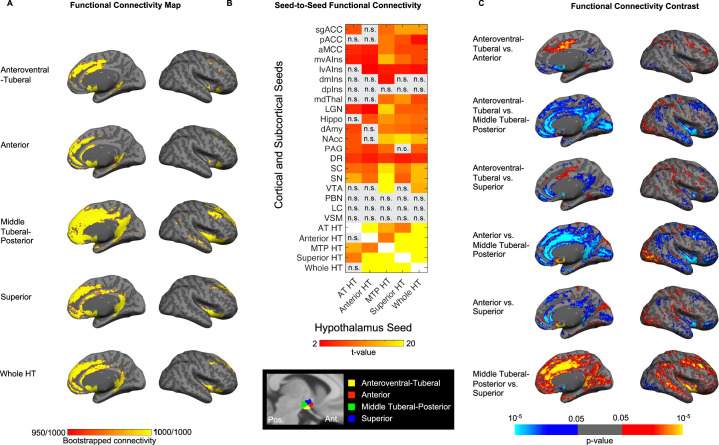
Intrinsic connectivity of the hypothalamus and its subregions within the allostatic-interoceptive system. **a**, Bootstrapped connectivity maps obtained from resampling 80% of the sample (*n* = 72) 1000 times. **b**, Seed-to-seed connectivity strength between hypothalamic subregions and all other seeds (two-tailed t-test, *p* < 0.05, uncorrected; *n* = 90; white color indicates correlation = 1 and gray color indicates subthreshold correlations; *n* = 90). **c**, Contrasts obtained by paired-sample t-tests between subregional maps (two-tailed t-test, *p* < 0.05, uncorrected). The middle tuberal-posterior and superior communities showed more robust and extensive connectivity to midcingulate cortex and ventral mid insula than the anteroventral-tuberal and anterior communities, and the middle tuberal-posterior community further showed more extensive connectivity to posterior cingulate cortex and posterior insula. aMCC: anterior midcingulate cortex; AT HT: anteroventral-tuberal hypothalamus; dAmy: dorsal amygdala; dmIns: dorsal mid insula; dpIns: dorsal posterior insula; DR: dorsal raphe; hippo: hippocampus; HT: hypothalamus; LC: locus coeruleus; LGN: lateral geniculate nucleus; lvAIns: lateral ventral anterior insula; mdThal: mediodorsal thalamus; MTP HT: middle tuberal-posterior hypothalamus; mvAIns: medial ventral anterior insula; NAcc: nucleus accumbens; pACC: pregenual anterior cingulate cortex; PAG: periaqueductal gray; PBN: parabrachial nucleus; PCC: posterior cingulate cortex; SC: superior colliculus; sgACC: subgenual anterior cingulate cortex; SN: substantia nigra; VSM: medullary viscero-sensory-motor nuclei complex, including the nucleus tractus solitarius, dorsal motor nucleus of the vagus, nucleus ambiguus, and hypoglossal nucleus; VTA: ventral tegmental area.

With our newly delineated subcortical seeds^45,48,83^, we also assessed subcortico-subcortical connectivity by visually inspecting subcortical maps of the subcortical seeds to confirm topography (Fig. 6a and see Extended Data Fig. 3 for bootstrapped maps identifying connectivity surviving *P* < 0.05 in more than 95% of subsampled analyses) and by calculating functional connectivity between all subcortical seeds to quantify strength of connection (Fig. 6b). Again, this analysis presented considerable challenges with 3 Tesla scanning as discussed in ref. ^19^. Subcortico-subcortical functional connectivity within the allostatic–interoceptive system confirmed 96% of the monosynaptic connections documented in published tract-tracing studies of nonhuman animals (see Supplementary Table 1 and references therein). There were three exceptions as follows: we did not observe significant, positive functional connectivity between hypothalamus and PBN, hypothalamus and LC, or hypothalamus and VSM, despite known anatomical connections (Supplementary Table 1). In one case, averaged time courses between the VSM and NAcc seeds did not correlate significantly (Fig. 6b, gray square in matrix), but bilateral NAcc clusters could nonetheless be observed in the VSM-seeded map. As in the subcortico-cortical maps, such discrepancies can result from noisy signals within an ROI or specific subportions of an ROI showing significant connectivity. Seed-to-seed connectivity strength between PAG subregions and other subcortical ROIs is displayed in Extended Data Fig. 4. Seed-to-seed connectivity strength between hippocampal subregions and other subcortical ROIs is displayed in Extended Data Fig. 5. Seed-to-seed connectivity strength between layers of the SC and other subcortical ROIs is displayed in Extended Data Fig. 6. Seed-to-seed connectivity strength between hypothalamus subregions and other subcortical ROIs is displayed in Extended Data Fig. 7. Conjoined binarized subcortical discovery maps (*P* < 0.05) indicated that all but four subcortical seeds showed overlapping connectivity: connecting regions were identified in the mdThal, LGN, hippocampus, dAmy, NAcc, PAG, DR, SC, SN and VTA but hypothalamus, PBN, LC and VSM showed less widespread and dense connectivity throughout subcortical seeds (Supplementary Table 3). The *k*-means clustering analysis (*k* = 3) on the subcortical discovery maps from subcortical seeds yielded an almost identical solution as their cortical connectivity maps.

### The allostatic–interoceptive system

**Fig. 7:**
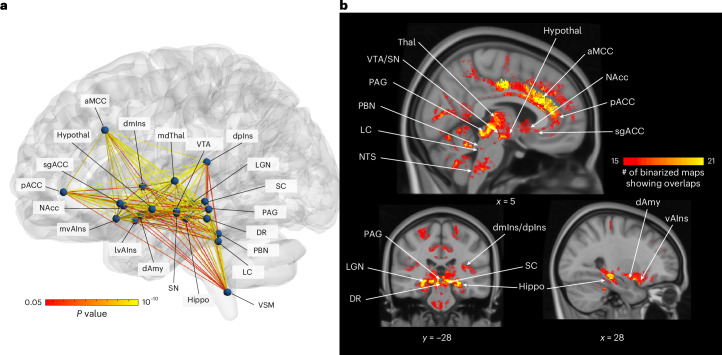
Summary of the allostatic–interoceptive system based on 7 Tesla fMRI functional connectivity. **a**, Circuit diagram indicates dense within-system connectivity between the 21 cortical and subcortical seeds. All seeds are shown as spherical nodes located at their respective centers of gravity. Pairwise connectivity strengths between ROIs are shown as edges between nodes (two-tailed *t* test, ranging from *P* < 0.05 in red to *P* < 10^−10^ in yellow, uncorrected; *n* = 90). Nodes and edges in the glass brain were visualized using BrainNet Viewer^151^. **b**, Conjunction map shows the number of binarized maps (two-tailed *t* test, *P* < 0.05) that shared overlapping regions (ranging from 15 to 21, total number of cortical and subcortical seeds = 21).

We observed dense interconnectivity between all the cortical and subcortical seeds included in our analysis (Fig. 7a). Conjoined binarized discovery maps (*P* < 0.05) across both cortical and subcortical extents converged in the hypothesized allostatic–interoceptive system (Fig. 7b).

## Discussion

Ultrahigh-field 7 Tesla fMRI with 1.1-mm isotropic voxel resolution combined with newly delineated 7 Tesla brainstem and diencephalic parcellations^44–48^ revealed both cortical and subcortical components of an integrated allostatic–interoceptive system in humans. Our original study applying 3 Tesla fMRI^19^ used 10-min resting state scans in two subsamples of 270–280 participants each, as well as a third sample of *n* = 41, whereas the present study involved a greater duration of resting state scan time (30 min in total) in a sample of 90 participants. Using functional connectivity among 7 cortical ROIs and 14 subcortical ROIs in humans, we verified more than 96% of the anatomical connections identified in published tract-tracing studies of macaques and other nonhuman mammals. Our current 7 Tesla findings revealed reciprocal connectivity between sgACC/pACC and dmIns/dpIns regions that was previously unreported in 3 Tesla functional connectivity studies of the ACC^87–91^ and the insula^92–95^. The improvement in sgACC connectivity, in particular, was expected at 7 Tesla, as this region is a part of the medial/orbital surface that is typically susceptible to low SNR, partial volume effects and physiological aliasing. In the current study, these effects were mitigated by higher resolution image acquisition at 7 Tesla, minimal smoothing and more precise nuisance regression using signals from individual ventricles. We also expanded observations of the subcortical extents of the system. Several subcortical nodes (that is, mdThal, LGN, hippocampus, dAmy, NAcc, SC, SN and VTA) showed robust connectivity with all cortical nodes, whereas the smaller brainstem nuclei (that is, PAG, DR, PBN, LC and VSM (including the NTS)) showed weaker but reliable connectivity to these nodes, consistent with other studies that examined a subset of the nodes as seeds at 3 Tesla (for example, ref. ^49^) and 7 Tesla (for example, refs. ^30,31,96,97^). We also observed reliable connectivity between regions that had not been previously documented as having monosynaptic connections in tract-tracing studies. For example, the LGN has virtually no monosynaptic connectivity with cortical nodes of the allostatic–interoceptive system according to the tract-tracing studies (except for modest projections to the pACC^69^), yet we observed reliable functional connectivity between the LGN and the aMCC, medial vAIns and pACC. The LGN receives interoceptive input^67^ and there is some evidence that interoceptive signals gate visual sensory sampling^98^, suggesting that LGN functional connectivity with other nodes of the allostatic–interoceptive system reflects polysynaptic connections that are functionally meaningful. In our study, the observation of a broad allostatic–interoceptive system is consistent with the confirmed monosynaptic connections between the a priori ROIs and the understanding that functional connectivity may reflect both monosynaptic and polysynaptic connections^99^.

The connecting ‘hub’ regions of the allostatic–interoceptive system observed at 7 Tesla covered *all* hypothesized cortical regions of interest, including the full extent of primary interoceptive cortex (dpIns, dmIns^15^) and the primary visceromotor regions (vAIns, sgACC, pACC and aMCC^100^). Several other connecting ‘hub’ regions (MCC, PCC, IFG, PHG, STG) were also observed and we confirmed their anatomical connections to documented allostatic regions in nonhuman animals^2,84,101–104^. The remaining connecting regions (that is, MFG, SFG, isthmus of the cingulate, cuneus) have not been documented as having monosynaptic anatomical connections to our subcortical and cortical seed regions; their functional connectivity may reflect polysynaptic connections or new connections in humans. Notably, most of the additional connecting regions observed at 7 Tesla (that is, pACC, PCC, isthmus cingulate, SFG, MFG and mid insula; except the sgACC) belong to the ‘rich club’ (the most densely interconnected regions in the cortex and thought to serve as the ‘backbone’ that synchronizes neural communication throughout the brain^105^), consistent with the hypothesized central role of the allostatic–interoceptive system as a high-capacity ‘backbone’ for integrating information across the entire brain^106^.

The results of this study have several important functional implications. First, several brain regions within the allostatic–interoceptive network likely have a crucial role in coordinating and regulating the body’s systems, although they are also involved in other psychological phenomena. For example, the SC is typically studied for visuomotor functioning in humans but has been shown to be important for approach and avoidance behavior, as well as the accompanying changes in visceromotor activity in nonhuman mammals (for example, refs. ^62,107^) through anatomical connections to ACC^50^ and hypothalamus^51^. Similarly, the hippocampus is usually considered central to memory function, but evidence from nonhuman animals indicates that the hippocampus also has a role in the regulation of feeding behaviors and in interoception-related reward signals^108–111^. There is also circumstantial evidence that interoceptive signals, relayed from the vagus nerve to the hippocampus via the NTS and septal nuclei, may have a role in event segmentation^112,113^. Furthermore, the LGN is usually considered part of the visual pathway that relays visual information from the retina and the cerebral cortex. However, the current functional connectivity findings are consistent with tract-tracing evidence showing LGN’s monosynaptic connections with cortical (for example, pACC^69^) and subcortical visceromotor structures (for example, hypothalamus^68^, PAG^52^ and PBN^114^), suggesting a role for facilitating communication among brain structures implicated in bodily regulation, in addition to its role in integrating interoceptive and visual signals^39^. The broad functional connectivity profile of the LGN is also consistent with evidence of tracts between the LGN and other hypothesized regions of the allostatic–interoceptive network, such as the hippocampus, amygdala, DR, SC, SN and LC (Supplementary Table 1).

Second, both the default mode and salience networks have been functionally implicated in cardiovascular regulation as well as in other aspects of allostasis^9,115,116^, and they have also been implicated in mental and physical illnesses and their comorbidities. Not surprisingly, psychiatric illnesses (for example, depression^117^, schizophrenia^118^), neurodevelopmental illnesses (for example, sensory processing disorder/autism spectrum disorder^119^), neurodegenerative illnesses (for example, dementia/Alzheimer’s disease^120^, Parkinson’s disease^121^) and physical illnesses (for example, heart disease^122^, chronic pain^123^) present with symptoms related to altered interoception or visceromotor control, and some of these symptoms are transdiagnostic^124,125^. Moreover, interoceptive and visceromotor symptomatology is often accompanied by altered neurobiology (for example, volume, structural connectivity, functional connectivity, evoked potential, task activation) in the allostatic–interoceptive system (for example, depression^126^, autism^127^, dementia^33,120^, chronic pain^128^). In addition, there is evidence showing that psychological therapies targeting interoceptive processes^129^ and neuromodulations targeting distributed regions within the allostatic–interoceptive system^130,131^ may be effective transdiagnostic interventions. Taken together, these findings suggest that altered function of the allostatic–interoceptive system may be a transdiagnostic feature of mental and physical illness that holds promising clinical utility. More fundamentally, the system identified in this paper provides a scientific tool for integrating studies across psychological and illness domains in a manner that will speed discovery, the accumulation of knowledge and, potentially, strategies for more effective treatments and prevention.

Finally, the findings reported here are consistent with the growing body of evidence that a number of subcortical and cortical brain regions are important during both the regulation of bodily functions and during cognitive phenomena, calling into question their functional segregation^132–134^. Our findings suggest that the default mode and salience networks may be concurrently coordinating, regulating and representing organs and tissues of the internal milieu at the same time that they are engaged in a wide range of tasks spanning cognitive, perceptual, emotion and action domains (Fig. 5 in ref. ^19^). Therefore, our results, when situated in the broader published literature, suggest that the default mode and salience networks create a highly connected functional ensemble for integrating information across the brain, with interoceptive and allostatic signaling at the core. Regulation of the body has been largely ignored in the neuroscientific study of the mind, in part, because much of interoceptive modeling occurs outside of human awareness^18,125^.

Several limitations within the current study should be addressed in future studies. First, we did not validate the connectivity strength within the allostatic–interoceptive network against signal-based measures of interoception (for example, heart-beat evoked potentials), although there is growing evidence that, even at rest, limbic regions of the brain continually issue allostatic control signals and there should be synchronous relationships between resting state BOLD signals and electrical signals from visceromotor movements^135^. Second, we did not fully monitor participants’ wakefulness (for example, through video recording) during the three 10-min resting state scans. The default mode and salience networks are present during sleep^136^, although the strength of within-network functional connectivity has been shown to vary (with evidence of both stronger and weaker connectivity) as a function of wakefulness^137–140^. Third, susceptibility-induced field inhomogeneities and distortions are more pronounced in 7 Tesla echo planar imaging scans. Although we applied bias field correction on the T1-weighted echo planar imaging to help mitigate these effects, further systematic investigation is needed to evaluate their impact on functional connectivity analysis, particularly in regions with high susceptibility, such as the orbitofrontal cortex. Finally, we did not map every relevant subcortical area that may be involved in allostasis or interoception. For example, opportunities for further research include septal nuclei (with direct projections to limbic regions such as the hippocampus and implicated in temporal control of neurons that make up the allostatic–interoceptive network^141,142^), circumventricular organs (for example, area postrema with unique access to peripheral signaling molecules through its permeable blood-brain barrier^143,144^) and motor brainstem nuclei (for example, dorsal motor nucleus of the vagus and nucleus ambiguus whose neurons give rise to the efferent vagus nerve^145,146^). The cerebellum is also likely involved in allostasis and interoception^147,148^.

## Methods

### Participants and MRI acquisition

We recruited 140 native English-speaking adults with normal or corrected-to-normal vision and no history of neurological or psychiatric conditions. All participants provided written informed consent and were compensated in accordance with the guidelines set by the institutional review board of Massachusetts General Hospital. A total of 50 participants were excluded from the current analysis (19 withdrew before the MRI session, 3 withdrew during MRI acquisition due to discomfort, 6 did not complete scans due to online scan reconstruction failure, 3 did not complete scans due to time constraint, 4 were excluded due to other technical issues during acquisition, 10 were excluded due to scanner sequence error, 4 were excluded due to corrupted MRI data that could not be processed and 1 was excluded due to excessive artifacts in the structural scan). This resulted in a final sample of 90 participants (26.9 ± 6.2 years old; 40 females, 50 males). MRI data were acquired using a 7 Tesla scanner (Magnetom, Siemens Healthineers) with a 32-channel phased-array head coil and personalized padding to achieve a tight fit. Participants completed a structural scan, three resting state scans of 10 min each, three diffusion-weighted scans, as well as other tasks unrelated to the current analysis. At the beginning of each resting state scan, participants were instructed to keep their eyes open and indicated their readiness to start the scan by pressing a button. MRI parameters are detailed in Supplementary Note.

### Preprocessing of fMRI data

The preprocessing pipeline began with reorientation, slice timing correction, concatenation of all three resting state runs, coregistration to the structural image and motion correction (framewise displacement mean = 0.17, s.d. = 0.14, with 98.7% of frames showing subvoxel motion^152^). We then conducted nuisance regression to remove physiological noise due to motion (six parameters measuring rotation and translation), as well as due to non-BOLD effects evaluated in the white matter, ventricular cerebrospinal fluid and the cerebral aqueduct. We then conducted temporal filtering and normalization. Finally, we performed conversion to Freesurfer orientation/dimensions, detrending, spatial smoothing (1.25 mm) and resampling to cortical surfaces. Preprocessing details are provided in Supplementary Note.

### Functional connectivity analysis

Seven cortical seeds (4-mm-radius spheres) were defined based on the previous fMRI studies of interoception using the procedure outlined in ref. ^19^. The 14 subcortical seeds were defined based on the Brainstem Navigator toolkit (https://www.nitrc.org/projects/brainstemnavig/; for example, ref. ^96^), CANLAB Combined Atlas 2018 (github.com/canlab) and Freesurfer segmentation (for example, ref. ^153^). See Supplementary Note for details about seed definition. We randomly resampled 80% of the sample (*n* = 72) 1,000 times. In each iteration, for each seed, we estimated cortical connectivity using Freesurfer-based analysis procedure as outlined in ref. ^19^. This yielded final group maps that showed regions whose fluctuations significantly correlated with the seed’s fMRI time series, which were binarized to retain positive connectivity surviving the threshold of *P* < .05 and summed across 1,000 iterations to obtain ‘bootstrapped connectivity’ maps. We also quantified seed-to-seed functional connectivity by computing Pearson’s correlation coefficient between all pairs of ROIs and applying Fisher’s *r*-to-*z* transform. Significance at the group level was assessed with a two-tailed one-sample *t* test.

### Connecting regions and *k*-means cluster analysis

To visualize the connecting ‘hub’ regions, we combined binarized functional connectivity maps (*P* < 0.05) for all seeds. To replicate the previously discovered two-subsystem distinction within the allostatic–interoceptive network^19^, we first computed a similarity matrix capturing pairwise *η*^2^ (ref. ^154^) between the unthresholded bootstrapped group maps of cortical seeds and then applied *k*-means clustering algorithm (*k*-means, MATLAB) with a range of *k* values between 2 and 10 (for each *k*, we tested ten initializations with new centroid positions, each with a maximum of 1,000 iterations to find the lowest local minimum for sum of distances). We evaluated the optimal *k* using the Calinski–Harabasz criterion^79^. To visualize each subsystem, we binarized the group connectivity maps (*P* < 0.05) and calculated the conjunction between maps within the same cluster.

### Reporting summary

Further information on research design is available in the Nature Portfolio Reporting Summary linked to this article.

## Online content

Any methods, additional references, Nature Portfolio reporting summaries, source data, extended data, supplementary information, acknowledgements, peer review information; details of author contributions and competing interests; and statements of data and code availability are available at 10.1038/s41593-025-02087-x.

## Supplementary information

Supplementary Information Supplementary Tables 1–3, Figs. 1 and 2 and Note.

Reporting Summary

## Data Availability

Raw and preprocessed data can be found at https://openneuro.org/datasets/ds005747.

## Code availability

Analysis outputs and codes can be found at https://github.com/jiahez/7-Tesla-Allostatic-Interoceptive-System.

## References

[1] Barbas, H. & Rempel-Clower, N. Cortical structure predicts the pattern of corticocortical connections. *Cereb. Cortex* **7**, 635–646 (1997). 9373019 10.1093/cercor/7.7.635

[2] Ongür, D., Ferry, A. T. & Price, J. L. Architectonic subdivision of the human orbital and medial prefrontal cortex. *J. Comp. Neurol.* **460**, 425–449 (2003). 12692859 10.1002/cne.10609

[3] Barrett, L. F. & Simmons, W. K. Interoceptive predictions in the brain. *Nat. Rev. Neurosci.* **16**, 419–429 (2015). 26016744 10.1038/nrn3950 PMC4731102

[4] Friston, K., FitzGerald, T., Rigoli, F., Schwartenbeck, P. & Pezzulo, G. Active inference: a process theory. *Neural Comput.* **29**, 1–49 (2017). 27870614 10.1162/NECO_a_00912

[5] Hutchinson, J. B. & Barrett, L. F. The power of predictions: an emerging paradigm for psychological research. *Curr. Dir. Psychol. Sci.* **28**, 280–291 (2019). 31749520 10.1177/0963721419831992 PMC6867616

[6] Straka, H., Simmers, J. & Chagnaud, B. P. A new perspective on predictive motor signaling. *Curr. Biol.* **28**, R232–R243 (2018). 29510116 10.1016/j.cub.2018.01.033

[7] Benarroch, E. E. The central autonomic network: functional organization, dysfunction, and perspective. *Mayo Clin. Proc.* **68**, 988–1001 (1993). 8412366 10.1016/s0025-6196(12)62272-1

[8] Gianaros, P. J. & Wager, T. D. Brain-body pathways linking psychological stress and physical health. *Curr. Dir. Psychol. Sci.* **24**, 313–321 (2015). 26279608 10.1177/0963721415581476 PMC4535428

[9] Valenza, G. et al. The central autonomic network at rest: uncovering functional MRI correlates of time-varying autonomic outflow. *Neuroimage* **197**, 383–390 (2019). 31055043 10.1016/j.neuroimage.2019.04.075

[10] Valenza, G., Ciò, F. D., Toschi, N. & Barbieri, R. Sympathetic and parasympathetic central autonomic networks. *Imaging Neurosci. (Camb.)* **2**, imag_2_00094 (2024). 40800371 10.1162/imag_a_00094 PMC12224469

[11] Valenza, G., Passamonti, L., Duggento, A., Toschi, N. & Barbieri, R. Uncovering complex central autonomic networks at rest: a functional magnetic resonance imaging study on complex cardiovascular oscillations. *J. R. Soc. Interface* **17**, 20190878 (2020). 32183642 10.1098/rsif.2019.0878 PMC7115224

[12] Sterling, P. Allostasis: a model of predictive regulation. *Physiol. Behav.* **106**, 5–15 (2012). 21684297 10.1016/j.physbeh.2011.06.004

[13] Sterling, P. & Laughlin, S. *Principles of Neural Design* (MIT Press, 2015).

[14] Katsumi, Y., Theriault, J. E., Quigley, K. S. & Barrett, L. F. Allostasis as a core feature of hierarchical gradients in the human brain. *Netw. Neurosci.* **6**, 1010–1031 (2022). 38800458 10.1162/netn_a_00240 PMC11117115

[15] Craig, A. D. How do you feel? Interoception: the sense of the physiological condition of the body. *Nat. Rev. Neurosci.* **3**, 655–666 (2002). 12154366 10.1038/nrn894

[16] Craig, A. D. *How Do You Feel?: An Interoceptive Moment With Your Neurobiological Self* (Princeton University Press, 2014).

[17] Critchley, H. D. & Harrison, N. A. Visceral influences on brain and behavior. *Neuron* **77**, 624–638 (2013). 23439117 10.1016/j.neuron.2013.02.008

[18] Quigley, K. S., Kanoski, S., Grill, W. M., Barrett, L. F. & Tsakiris, M. Functions of interoception: from energy regulation to experience of the self. *Trends Neurosci.* **44**, 29–38 (2021). 33378654 10.1016/j.tins.2020.09.008 PMC7780233

[19] Kleckner, I. R. et al. Evidence for a large-scale brain system supporting allostasis and interoception in humans. *Nat. Hum. Behav.* **1**, 0069 (2017). 28983518 10.1038/s41562-017-0069 PMC5624222

[20] Berntson, G. G. & Khalsa, S. S. Neural circuits of interoception. *Trends Neurosci.* **44**, 17–28 (2021). 33378653 10.1016/j.tins.2020.09.011 PMC8054704

[21] Evrard, H. C. The organization of the primate insular cortex. *Front. Neuroanat.* **13**, 43 (2019). 31133822 10.3389/fnana.2019.00043 PMC6517547

[22] Gianaros, P. J. & Sheu, L. K. A review of neuroimaging studies of stressor-evoked blood pressure reactivity: emerging evidence for a brain-body pathway to coronary heart disease risk. *Neuroimage* **47**, 922–936 (2009). 19410652 10.1016/j.neuroimage.2009.04.073 PMC2743251

[23] Harper, R. M. et al. fMRI responses to cold pressor challenges in control and obstructive sleep apnea subjects. *J. Appl. Physiol.* **94**, 1583–1595 (2003). 12514164 10.1152/japplphysiol.00881.2002

[24] Satpute, A. B., Kragel, P. A., Barrett, L. F., Wager, T. D. & Bianciardi, M. Deconstructing arousal into wakeful, autonomic and affective varieties. *Neurosci. Lett.* **693**, 19–28 (2019). 29378297 10.1016/j.neulet.2018.01.042 PMC6068010

[25] Wager, T. D. et al. Brain mediators of cardiovascular responses to social threat: part I: reciprocal dorsal and ventral sub-regions of the medial prefrontal cortex and heart-rate reactivity. *Neuroimage* **47**, 821–835 (2009). 19465137 10.1016/j.neuroimage.2009.05.043 PMC3275821

[26] Zunhammer, M., Spisák, T., Wager, T. D. & Bingel, U. Meta-analysis of neural systems underlying placebo analgesia from individual participant fMRI data. *Nat. Commun.* **12**, 1391 (2021). 33654105 10.1038/s41467-021-21179-3 PMC7925520

[27] Sclocco, R., Beissner, F., Bianciardi, M., Polimeni, J. R. & Napadow, V. Challenges and opportunities for brainstem neuroimaging with ultrahigh field MRI. *Neuroimage* **168**, 412–426 (2018). 28232189 10.1016/j.neuroimage.2017.02.052 PMC5777900

[28] Newton, A. T., Rogers, B. P., Gore, J. C. & Morgan, V. L. Improving measurement of functional connectivity through decreasing partial volume effects at 7 T. *Neuroimage* **59**, 2511–2517 (2012). 21925611 10.1016/j.neuroimage.2011.08.096 PMC3254819

[29] Bandettini, P. A., Bowtell, R., Jezzard, P. & Turner, R. Ultrahigh field systems and applications at 7 T and beyond: progress, pitfalls, and potential. *Magn. Reson. Med.* **67**, 317–321 (2012). 22083719 10.1002/mrm.23151 PMC3265677

[30] Cauzzo, S. et al. Functional connectome of brainstem nuclei involved in autonomic, limbic, pain and sensory processing in living humans from 7 Tesla resting state fMRI. *Neuroimage* **250**, 118925 (2022). 35074504 10.1016/j.neuroimage.2022.118925 PMC8885980

[31] Hansen, J. Y. et al. Integrating brainstem and cortical functional architectures. *Nat. Neurosci.* **27**, 2500–2511 (2024). 39414973 10.1038/s41593-024-01787-0 PMC11614745

[32] Migeot, J. et al. Allostatic-interoceptive anticipation of social rejection. *Neuroimage* **276**, 120200 (2023). 37245560 10.1016/j.neuroimage.2023.120200 PMC11163516

[33] Birba, A. et al. Allostatic-interoceptive overload in frontotemporal dementia. *Biol. Psychiatry* **92**, 54–67 (2022). 35491275 10.1016/j.biopsych.2022.02.955 PMC11184918

[34] Ruiz-Rizzo, A. L. et al. Human subsystems of medial temporal lobes extend locally to amygdala nuclei and globally to an allostatic-interoceptive system. *Neuroimage* **207**, 116404 (2020). 31783114 10.1016/j.neuroimage.2019.116404

[35] Alvarez, G. M., Rudolph, M. D., Cohen, J. R. & Muscatell, K. A. Lower socioeconomic position is associated with greater activity in and integration within an allostatic-interoceptive brain network in response to affective stimuli. *J. Cogn. Neurosci.* **34**, 1906–1927 (2022). 35139207 10.1162/jocn_a_01830

[36] Barrett, L. F. & Satpute, A. B. Large-scale brain networks in affective and social neuroscience: towards an integrative functional architecture of the brain. *Curr. Opin. Neurobiol.* **23**, 361–372 (2013). 23352202 10.1016/j.conb.2012.12.012 PMC4119963

[37] Anderson, M. L. *After Phrenology: Neural Reuse and the Interactive Brain* (MIT Press, 2014).

[38] Yeo, B. T. T. et al. Functional specialization and flexibility in human association cortex. *Cereb. Cortex* **25**, 3654–3672 (2015). 25249407 10.1093/cercor/bhu217 PMC4598819

[39] Azzalini, D., Rebollo, I. & Tallon-Baudry, C. Visceral signals shape brain dynamics and cognition. *Trends Cogn. Sci.* **23**, 488–509 (2019). 31047813 10.1016/j.tics.2019.03.007

[40] Raut, R. V. et al. Global waves synchronize the brain’s functional systems with fluctuating arousal. *Sci. Adv.* **7**, eabf2709 (2021). 34290088 10.1126/sciadv.abf2709 PMC8294763

[41] Rebollo, I. & Tallon-Baudry, C. The sensory and motor components of the cortical hierarchy are coupled to the rhythm of the stomach during rest. *J. Neurosci.* **42**, 2205–2220 (2022). 35074866 10.1523/JNEUROSCI.1285-21.2021 PMC8936619

[42] Polimeni, J. R. et al. Reducing sensitivity losses due to respiration and motion in accelerated echo planar imaging by reordering the autocalibration data acquisition. *Magn. Reson. Med.* **75**, 665–679 (2016). 25809559 10.1002/mrm.25628 PMC4580494

[43] Boubela, R. N. et al. fMRI measurements of amygdala activation are confounded by stimulus correlated signal fluctuation in nearby veins draining distant brain regions. *Sci. Rep.* **5**, 10499 (2015). 25994551 10.1038/srep10499 PMC4440210

[44] Bianciardi, M. et al. Toward an in vivo neuroimaging template of human brainstem nuclei of the ascending arousal, autonomic, and motor systems. *Brain Connect* **5**, 597–607 (2015). 26066023 10.1089/brain.2015.0347 PMC4684653

[45] García-Gomar, M. G. et al. In vivo probabilistic structural atlas of the inferior and superior colliculi, medial and lateral geniculate nuclei and superior olivary complex in humans based on 7 Tesla MRI. *Front. Neurosci.* **13**, 764 (2019). 31440122 10.3389/fnins.2019.00764 PMC6694208

[46] García-Gomar, M. G. et al. Disruption of brainstem structural connectivity in REM sleep behavior disorder using 7 Tesla magnetic resonance imaging. *Mov. Disord.* **37**, 847–853 (2022). 34964520 10.1002/mds.28895 PMC9018552

[47] Singh, K. et al. Probabilistic template of the lateral parabrachial nucleus, medial parabrachial nucleus, vestibular nuclei complex, and medullary viscero-sensory-motor nuclei complex in living humans from 7 Tesla MRI. *Front. Neurosci.* **13**, 1425 (2020). 32038134 10.3389/fnins.2019.01425 PMC6989551

[48] Singh, K., García-Gomar, M. G. & Bianciardi, M. Probabilistic atlas of the mesencephalic reticular formation, isthmic reticular formation, microcellular tegmental nucleus, ventral tegmental area nucleus complex, and caudal–rostral linear raphe nucleus complex in living humans from 7 Tesla magnetic resonance imaging. *Brain Connect.* **11**, 613–623 (2021). 33926237 10.1089/brain.2020.0975 PMC8817713

[49] Bär, K.-J. et al. Functional connectivity and network analysis of midbrain and brainstem nuclei. *Neuroimage* **134**, 53–63 (2016). 27046112 10.1016/j.neuroimage.2016.03.071

[50] Harting, J. K., Huerta, M. F., Hashikawa, T. & van Lieshout, D. P. Projection of the mammalian superior colliculus upon the dorsal lateral geniculate nucleus: organization of tectogeniculate pathways in nineteen species. *J. Comp. Neurol.* **304**, 275–306 (1991). 1707899 10.1002/cne.903040210

[51] Benevento, L. A. & Fallon, J. H. The ascending projections of the superior colliculus in the rhesus monkey (*Macaca mulatta*). *J. Comp. Neurol.* **160**, 339–361 (1975). 1112928 10.1002/cne.901600306

[52] Beitz, A. J. The organization of afferent projections to the midbrain periaqueductal gray of the rat. *Neuroscience* **7**, 133–159 (1982). 7078723 10.1016/0306-4522(82)90157-9

[53] Card, J. P. & Moore, R. Y. Organization of lateral geniculate-hypothalamic connections in the rat. *J. Comp. Neurol.* **284**, 135–147 (1989). 2754028 10.1002/cne.902840110

[54] Mikkelsen, J. D. A neuronal projection from the lateral geniculate nucleus to the lateral hypothalamus of the rat demonstrated with *Phaseolus vulgaris* leucoagglutinin tracing. *Neurosci. Lett.* **116**, 58–63 (1990). 1701866 10.1016/0304-3940(90)90386-n

[55] Uhlrich, D. J., Cucchiaro, J. B. & Sherman, S. M. The projection of individual axons from the parabrachial region of the brain stem to the dorsal lateral geniculate nucleus in the cat. *J. Neurosci.* **8**, 4565–4575 (1988). 2848936 10.1523/JNEUROSCI.08-12-04565.1988 PMC6569552

[56] Fillinger, C., Yalcin, I., Barrot, M. & Veinante, P. Efferents of anterior cingulate areas 24a and 24b and midcingulate areas 24aʹ and 24bʹ in the mouse. *Brain Struct. Funct.* **223**, 1747–1778 (2018). 29209804 10.1007/s00429-017-1585-x

[57] Rieck, R. W., Huerta, M. F., Harting, J. K. & Weber, J. T. Hypothalamic and ventral thalamic projections to the superior colliculus in the cat. *J. Comp. Neurol.* **243**, 249–265 (1986). 3944279 10.1002/cne.902430208

[58] Fallon, J. H. & Moore, R. Y. Superior colliculus efferents to the hypothalamus. *Neurosci. Lett.* **14**, 265–270 (1979). 93726 10.1016/0304-3940(79)96159-7

[59] Wiberg, M. Reciprocal connections between the periaqueductal gray matter and other somatosensory regions of the cat mid brain: a possible mechanism of pain inhibition. *Ups. J. Med. Sci.* **97**, 37–47 (1992). 1523733 10.3109/03009739209179280

[60] Gandhi, N. J. & Katnani, H. A. Motor functions of the superior colliculus. *Annu. Rev. Neurosci.* **34**, 205–231 (2011). 21456962 10.1146/annurev-neuro-061010-113728 PMC3641825

[61] Isa, T., Marquez-Legorreta, E., Grillner, S. & Scott, E. K. The tectum/superior colliculus as the vertebrate solution for spatial sensory integration and action. *Curr. Biol.* **31**, R741–R762 (2021). 34102128 10.1016/j.cub.2021.04.001 PMC8190998

[62] Keay, K. A., Redgrave, P. & Dean, P. Cardiovascular and respiratory changes elicited by stimulation of rat superior colliculus. *Brain Res. Bull.* **20**, 13–26 (1988). 3277692 10.1016/0361-9230(88)90004-4

[63] Sahibzada, N., Dean, P. & Redgrave, P. Movements resembling orientation or avoidance elicited by electrical stimulation of the superior colliculus in rats. *J. Neurosci.* **6**, 723–733 (1986). 3958791 10.1523/JNEUROSCI.06-03-00723.1986 PMC6568468

[64] Damasio, A. & Carvalho, G. B. The nature of feelings: evolutionary and neurobiological origins. *Nat. Rev. Neurosci.* **14**, 143–152 (2013). 23329161 10.1038/nrn3403

[65] Damasio, A., Damasio, H. & Tranel, D. Persistence of feelings and sentience after bilateral damage of the insula. *Cereb. Cortex* **23**, 833–846 (2013). 22473895 10.1093/cercor/bhs077 PMC3657385

[66] Erişir, A., Horn, S. C. V., Bickford, M. E. & Sherman, S. M. Immunocytochemistry and distribution of parabrachial terminals in the lateral geniculate nucleus of the cat: a comparison with corticogeniculate terminals. *J. Comp. Neurol.* **377**, 535–549 (1997). 9007191

[67] Erişir, A., van Horn, S. C. & Sherman, S. M. Relative numbers of cortical and brainstem inputs to the lateral geniculate. *Proc. Natl Acad. Sci. USA* **94**, 1517–1520 (1997). 9037085 10.1073/pnas.94.4.1517 PMC19823

[68] Moore, R. Y., Weis, R. & Moga, M. M. Efferent projections of the intergeniculate leaflet and the ventral lateral geniculate nucleus in the rat. *J. Comp. Neurol.* **420**, 398–418 (2000). 10754510 10.1002/(sici)1096-9861(20000508)420:3<398::aid-cne9>3.0.co;2-9

[69] Morin, L. P. & Blanchard, J. H. Forebrain connections of the hamster intergeniculate leaflet: comparison with those of ventral lateral geniculate nucleus and retina. *Vis. Neurosci.* **16**, 1037–1054 (1999). 10614586 10.1017/s0952523899166069

[70] Coulombe, M.-A., Erpelding, N., Kucyi, A. & Davis, K. D. Intrinsic functional connectivity of periaqueductal gray subregions in humans: PAG subregional functional connectivity. *Hum. Brain Mapp.* **37**, 1514–1530 (2016). 26821847 10.1002/hbm.23117 PMC6867375

[71] Iglesias, J. E. et al. Bayesian longitudinal segmentation of hippocampal substructures in brain MRI using subject-specific atlases. *Neuroimage* **141**, 542–555 (2016). 27426838 10.1016/j.neuroimage.2016.07.020 PMC5026967

[72] Waguespack, H. F., Aguilar, B. L., Malkova, L. & Forcelli, P. A. Inhibition of the deep and intermediate layers of the superior colliculus disrupts sensorimotor gating in monkeys. *Front. Behav. Neurosci.* **14**, 610702 (2020). 33414708 10.3389/fnbeh.2020.610702 PMC7783047

[73] Parent, M. B., Higgs, S., Cheke, L. G. & Kanoski, S. E. Memory and eating: a bidirectional relationship implicated in obesity. *Neurosci. Biobehav. Rev.* **132**, 110–129 (2022). 34813827 10.1016/j.neubiorev.2021.10.051 PMC8816841

[74] Satpute, A. B. et al. Identification of discrete functional subregions of the human periaqueductal gray. *Proc. Natl Acad. Sci. USA* **110**, 17101–17106 (2013). 24082116 10.1073/pnas.1306095110 PMC3801046

[75] Morecraft, R. J. et al. Cytoarchitecture and cortical connections of the anterior cingulate and adjacent somatomotor fields in the rhesus monkey. *Brain Res. Bull.* **87**, 457–497 (2012). 22240273 10.1016/j.brainresbull.2011.12.005 PMC3295893

[76] Chiba, T., Kayahara, T. & Nakano, K. Efferent projections of infralimbic and prelimbic areas of the medial prefrontal cortex in the Japanese monkey, *Macaca fuscata*. *Brain Res.* **888**, 83–101 (2001). 11146055 10.1016/s0006-8993(00)03013-4

[77] Vogt, B. A. & Pandya, D. N. Cingulate cortex of the rhesus monkey: II. Cortical afferents. *J. Comp. Neurol.* **262**, 271–289 (1987). 3624555 10.1002/cne.902620208

[78] Pandya, D. N., van Hoesen, G. W. & Mesulam, M. M. Efferent connections of the cingulate gyrus in the rhesus monkey. *Exp. Brain Res.* **42**, 319–330 (1981). 6165607 10.1007/BF00237497

[79] Caliński, T. & Harabasz, J. A dendrite method for cluster analysis. *Commun. Stat.* **3**, 1–27 (1974).

[80] Dosenbach, N. U. F., Raichle, M. E. & Gordon, E. M. The brain’s action-mode network. *Nat. Rev. Neurosci.* **26**, 158–168 (2025). 39743556 10.1038/s41583-024-00895-x

[81] van den Heuvel, M. P. & Sporns, O. Rich-club organization of the human connectome. *J. Neurosci.* **31**, 15775–15786 (2011). 22049421 10.1523/JNEUROSCI.3539-11.2011 PMC6623027

[82] van den Heuvel, M. P., Kahn, R. S., Goñi, J. & Sporns, O. High-cost, high-capacity backbone for global brain communication. *Proc. Natl Acad. Sci. USA* **109**, 11372–11377 (2012). 22711833 10.1073/pnas.1203593109 PMC3396547

[83] Bianciardi, M. et al. In vivo functional connectome of human brainstem nuclei of the ascending arousal, autonomic, and motor systems by high spatial resolution 7-Tesla fMRI. *MAGMA* **29**, 451–462 (2016). 27126248 10.1007/s10334-016-0546-3 PMC4892960

[84] An, X., Bandler, R., Ongür, D. & Price, J. L. Prefrontal cortical projections to longitudinal columns in the midbrain periaqueductal gray in macaque monkeys. *J. Comp. Neurol.* **401**, 455–479 (1998). 9826273

[85] Jasmin, L., Granato, A. & Ohara, P. T. Rostral agranular insular cortex and pain areas of the central nervous system: a tract-tracing study in the rat. *J. Comp. Neurol.* **468**, 425–440 (2004). 14681935 10.1002/cne.10978

[86] Liu, X. et al. The superior colliculus: cell types, connectivity, and behavior. *Neurosci. Bull.* **38**, 1519–1540 (2022). 35484472 10.1007/s12264-022-00858-1 PMC9723059

[87] Chase, H. W., Grace, A. A., Fox, P. T., Phillips, M. L. & Eickhoff, S. B. Functional differentiation in the human ventromedial frontal lobe: a data-driven parcellation. *Hum. Brain Mapp.* **41**, 3266–3283 (2020). 32314470 10.1002/hbm.25014 PMC7375078

[88] Jin, F., Zheng, P., Liu, H., Guo, H. & Sun, Z. Functional and anatomical connectivity-based parcellation of human cingulate cortex. *Brain Behav.* **8**, e01070 (2018). 30039643 10.1002/brb3.1070 PMC6085915

[89] Palomero-Gallagher, N. et al. Human pregenual anterior cingulate cortex: structural, functional, and connectional heterogeneity. *Cereb. Cortex* **29**, 2552–2574 (2019). 29850806 10.1093/cercor/bhy124 PMC6519696

[90] Rolls, E. T. et al. Functional connectivity of the anterior cingulate cortex in depression and in health. *Cereb. Cortex* **29**, 3617–3630 (2019). 30418547 10.1093/cercor/bhy236

[91] Yu, C. et al. Functional segregation of the human cingulate cortex is confirmed by functional connectivity based neuroanatomical parcellation. *Neuroimage* **54**, 2571–2581 (2011). 21073967 10.1016/j.neuroimage.2010.11.018

[92] Cauda, F. et al. Functional connectivity of the insula in the resting brain. *Neuroimage* **55**, 8–23 (2011). 21111053 10.1016/j.neuroimage.2010.11.049

[93] Chang, L. J., Yarkoni, T., Khaw, M. W. & Sanfey, A. G. Decoding the role of the insula in human cognition: functional parcellation and large-scale reverse inference. *Cereb. Cortex* **23**, 739–749 (2013). 22437053 10.1093/cercor/bhs065 PMC3563343

[94] Deen, B., Pitskel, N. B. & Pelphrey, K. A. Three systems of insular functional connectivity identified with cluster analysis. *Cereb. Cortex* **21**, 1498–1506 (2011). 21097516 10.1093/cercor/bhq186 PMC3116731

[95] Kelly, C. et al. A convergent functional architecture of the insula emerges across imaging modalities. *Neuroimage* **61**, 1129–1142 (2012). 22440648 10.1016/j.neuroimage.2012.03.021 PMC3376229

[96] Singh, K. et al. Functional connectome of arousal and motor brainstem nuclei in living humans by 7 Tesla resting-state fMRI. *Neuroimage* **249**, 118865 (2022). 35031472 10.1016/j.neuroimage.2021.118865 PMC8856580

[97] Groot, J. M. et al. Echoes from intrinsic connectivity networks in the subcortex. *J. Neurosci.* **43**, 6609–6618 (2023). 37562962 10.1523/JNEUROSCI.1020-23.2023 PMC10538587

[98] Ren, Q., Marshall, A. C., Kaiser, J. & Schütz-Bosbach, S. Multisensory integration of anticipated cardiac signals with visual targets affects their detection among multiple visual stimuli. *Neuroimage* **262**, 119549 (2022). 35940424 10.1016/j.neuroimage.2022.119549

[99] Bazinet, V., vos de Wael, R., Hagmann, P., Bernhardt, B. C. & Misic, B. Multiscale communication in cortico-cortical networks. *Neuroimage* **243**, 118546 (2021). 34478823 10.1016/j.neuroimage.2021.118546

[100] Ongür, D. & Price, J. L. The organization of networks within the orbital and medial prefrontal cortex of rats, monkeys and humans. *Cereb. Cortex* **10**, 206–219 (2000). 10731217 10.1093/cercor/10.3.206

[101] Demeter, S., Rosene, D. L. & van Hoesen, G. W. Interhemispheric pathways of the hippocampal formation, presubiculum, and entorhinal and posterior parahippocampal cortices in the rhesus monkey: the structure and organization of the hippocampal commissures. *J. Comp. Neurol.* **233**, 30–47 (1985). 3980771 10.1002/cne.902330104

[102] Kobayashi, Y. & Amaral, D. G. Macaque monkey retrosplenial cortex: III. Cortical efferents. *J. Comp. Neurol.* **502**, 810–833 (2007). 17436282 10.1002/cne.21346

[103] Olson, C. R. & Musil, S. Y. Topographic organization of cortical and subcortical projections to posterior cingulate cortex in the cat: evidence for somatic, ocular, and complex subregions. *J. Comp. Neurol.* **324**, 237–260 (1992). 1430331 10.1002/cne.903240207

[104] Risold, P. Y., Thompson, R. H. & Swanson, L. W. The structural organization of connections between hypothalamus and cerebral cortex. *Brain Res. Brain Res. Rev.* **24**, 197–254 (1997). 9385455 10.1016/s0165-0173(97)00007-6

[105] Van den Heuvel, M. P. & Sporns, O. An anatomical substrate for integration among functional networks in human cortex. *J. Neurosci.* **33**, 14489–14500 (2013). 24005300 10.1523/JNEUROSCI.2128-13.2013 PMC6618386

[106] Zhang, J. et al. Topography impacts topology: anatomically central areas exhibit a `high-level connector' profile in the human cortex. *Cereb. Cortex* **30**, 1357–1365 (2020). 31504277 10.1093/cercor/bhz171 PMC7132940

[107] Maior, R. S. et al. A role for the superior colliculus in the modulation of threat responsiveness in primates: toward the ontogenesis of the social brain. *Rev. Neurosci.* **23**, 697–706 (2012). 23001312 10.1515/revneuro-2012-0055

[108] Gauthier, J. L. & Tank, D. W. A dedicated population for reward coding in the hippocampus. *Neuron* **99**, 179–193 (2018). 30008297 10.1016/j.neuron.2018.06.008 PMC7023678

[109] Kanoski, S. E. & Grill, H. J. Hippocampus contributions to food intake control: mnemonic, neuroanatomical, and endocrine mechanisms. *Biol. Psychiatry* **81**, 748–756 (2017). 26555354 10.1016/j.biopsych.2015.09.011 PMC4809793

[110] Noble, E. E. et al. Hypothalamus-hippocampus circuitry regulates impulsivity via melanin-concentrating hormone. *Nat. Commun.* **10**, 4923 (2019). 31664021 10.1038/s41467-019-12895-y PMC6820566

[111] Suarez, A. N., Liu, C. M., Cortella, A. M., Noble, E. E. & Kanoski, S. E. Ghrelin and orexin interact to increase meal size through a descending hippocampus to hindbrain signaling pathway. *Biol. Psychiatry* **87**, 1001–1011 (2020). 31836175 10.1016/j.biopsych.2019.10.012 PMC7188579

[112] Shaffer, C., Barrett, L. F. & Quigley, K. S. Signal processing in the vagus nerve: hypotheses based on new genetic and anatomical evidence. *Biol. Psychol.* **182**, 108626 (2023). 37419401 10.1016/j.biopsycho.2023.108626 PMC10563766

[113] Brændholt, M. et al. Breathing in waves: understanding respiratory-brain coupling as a gradient of predictive oscillations. *Neurosci. Biobehav. Rev.* **152**, 105262 (2023). 37271298 10.1016/j.neubiorev.2023.105262

[114] Hughes, H. C. & Mullikin, W. H. Brainstem afferents to the lateral geniculate nucleus of the cat. *Exp. Brain Res.* **54**, 253–258 (1984). 6723845 10.1007/BF00236224

[115] Ruffle, J. K. et al. The autonomic brain: multi-dimensional generative hierarchical modelling of the autonomic connectome. *Cortex* **143**, 164–179 (2021). 34438298 10.1016/j.cortex.2021.06.012 PMC8500219

[116] De la Cruz, F. et al. Central autonomic network alterations in Anorexia nervosa following peripheral adrenergic stimulation. *Biol. Psychiatry Cogn. Neurosci. Neuroimaging* **8**, 720–730 (2023). 37055325 10.1016/j.bpsc.2022.12.009 PMC10285030

[117] Shaffer, C., Westlin, C., Quigley, K. S., Whitfield-Gabrieli, S. & Barrett, L. F. Allostasis, action, and affect in depression: insights from the theory of constructed emotion. *Annu. Rev. Clin. Psychol.* **18**, 553–580 (2022). 35534123 10.1146/annurev-clinpsy-081219-115627 PMC9247744

[118] Yao, B. & Thakkar, K. Interoception abnormalities in schizophrenia: a review of preliminary evidence and an integration with Bayesian accounts of psychosis. *Neurosci. Biobehav. Rev.* **132**, 757–773 (2022). 34823914 10.1016/j.neubiorev.2021.11.016

[119] Schauder, K. B., Mash, L. E., Bryant, L. K. & Cascio, C. J. Interoceptive ability and body awareness in autism spectrum disorder. *J. Exp. Child Psychol.* **131**, 193–200 (2015). 25498876 10.1016/j.jecp.2014.11.002 PMC4303499

[120] García-Cordero, I. et al. Feeling, learning from and being aware of inner states: interoceptive dimensions in neurodegeneration and stroke. *Philos. Trans. R. Soc. Lond. B Biol. Sci.* **371**, 20160006 (2016). 28080965 10.1098/rstb.2016.0006 PMC5062096

[121] Ricciardi, L. et al. Know thyself: exploring interoceptive sensitivity in Parkinson’s disease. *J. Neurol. Sci.* **364**, 110–115 (2016). 27084227 10.1016/j.jns.2016.03.019

[122] Yoris, A. et al. Multicentric evidence of emotional impairments in hypertensive heart disease. *Sci. Rep.* **10**, 14131 (2020). 32839479 10.1038/s41598-020-70451-x PMC7445248

[123] Di Lernia, D., Lacerenza, M., Ainley, V. & Riva, G. Altered interoceptive perception and the effects of interoceptive analgesia in musculoskeletal, primary, and neuropathic chronic pain conditions. *J. Pers. Med.* **10**, 201 (2020). 33138185 10.3390/jpm10040201 PMC7712753

[124] Bonaz, B. et al. Diseases, disorders, and comorbidities of interoception. *Trends Neurosci.* **44**, 39–51 (2021). 33378656 10.1016/j.tins.2020.09.009

[125] Khalsa, S. S. et al. Interoception and mental health: a roadmap. *Biol. Psychiatry Cogn. Neurosci. Neuroimaging* **3**, 501–513 (2018). 29884281 10.1016/j.bpsc.2017.12.004 PMC6054486

[126] Hamilton, J. P., Farmer, M., Fogelman, P. & Gotlib, I. H. Depressive rumination, the default-mode network, and the dark matter of clinical neuroscience. *Biol. Psychiatry* **78**, 224–230 (2015). 25861700 10.1016/j.biopsych.2015.02.020 PMC4524294

[127] Uddin, L. Q. et al. Salience network-based classification and prediction of symptom severity in children with autism. *JAMA Psychiatry* **70**, 869–879 (2013). 23803651 10.1001/jamapsychiatry.2013.104 PMC3951904

[128] Baliki, M. N., Mansour, A. R., Baria, A. T. & Apkarian, A. V. Functional reorganization of the default mode network across chronic pain conditions. *PLoS ONE* **9**, e106133 (2014). 25180885 10.1371/journal.pone.0106133 PMC4152156

[129] Nord, C. L. & Garfinkel, S. N. Interoceptive pathways to understand and treat mental health conditions. *Trends Cogn. Sci.* **26**, 499–513 (2022). 35466044 10.1016/j.tics.2022.03.004

[130] Bauer, C. C. C. et al. Real-time fMRI neurofeedback reduces auditory hallucinations and modulates resting state connectivity of involved brain regions: part 2: default mode network—preliminary evidence. *Psychiatry Res.* **284**, 112770 (2020). 32004893 10.1016/j.psychres.2020.112770 PMC7046150

[131] Zhang, J. et al. Reducing default mode network connectivity with mindfulness-based fMRI neurofeedback: a pilot study among adolescents with affective disorder history. *Mol. Psychiatry* **28**, 2540–2548 (2023). 36991135 10.1038/s41380-023-02032-z PMC10611577

[132] Cesario, J., Johnson, D. J. & Eisthen, H. L. Your brain is not an onion with a tiny reptile inside. *Curr. Dir. Psychol. Sci.* **29**, 255–260 (2020).

[133] Chanes, L. & Barrett, L. F. Redefining the role of limbic areas in cortical processing. *Trends Cogn. Sci.* **20**, 96–106 (2016). 26704857 10.1016/j.tics.2015.11.005 PMC4780414

[134] Nakai, T. & Nishimoto, S. Representations and decodability of diverse cognitive functions are preserved across the human cortex, cerebellum, and subcortex. *Commun. Biol.* **5**, 1245 (2022). 36376490 10.1038/s42003-022-04221-y PMC9663596

[135] Engelen, T., Solcà, M. & Tallon-Baudry, C. Interoceptive rhythms in the brain. *Nat. Neurosci.* **26**, 1670–1684 (2023). 37697110 10.1038/s41593-023-01425-1

[136] Houldin, E., Fang, Z., Ray, L. B., Owen, A. M. & Fogel, S. M. Toward a complete taxonomy of resting state networks across wakefulness and sleep: an assessment of spatially distinct resting state networks using independent component analysis. *Sleep* **42**, zsy235 (2019). 30476346 10.1093/sleep/zsy235

[137] Horovitz, S. G. et al. Decoupling of the brain’s default mode network during deep sleep. *Proc. Natl Acad. Sci. USA* **106**, 11376–11381 (2009). 19549821 10.1073/pnas.0901435106 PMC2708777

[138] Tagliazucchi, E. & Laufs, H. Decoding wakefulness levels from typical fMRI resting-state data reveals reliable drifts between wakefulness and sleep. *Neuron* **82**, 695–708 (2014). 24811386 10.1016/j.neuron.2014.03.020

[139] Titone, S. et al. Frequency-dependent connectivity in large-scale resting-state brain networks during sleep. *Eur. J. Neurosci.* **59**, 686–702 (2024). 37381891 10.1111/ejn.16080

[140] Korponay, C., Janes, A. C. & Frederick, B. B. Brain-wide functional connectivity artifactually inflates throughout functional magnetic resonance imaging scans. *Nat. Hum. Behav.* **8**, 1568–1580 (2024). 38898230 10.1038/s41562-024-01908-6 PMC11526723

[141] Tsanov, M. Differential and complementary roles of medial and lateral septum in the orchestration of limbic oscillations and signal integration. *Eur. J. Neurosci.* **48**, 2783–2794 (2018). 29044802 10.1111/ejn.13746

[142] Takeuchi, Y. et al. The medial septum as a potential target for treating brain disorders associated with oscillopathies. *Front. Neural Circuits* **15**, 701080 (2021). 34305537 10.3389/fncir.2021.701080 PMC8297467

[143] Cottrell, G. T. & Ferguson, A. V. Sensory circumventricular organs: central roles in integrated autonomic regulation. *Regul. Pept.* **117**, 11–23 (2004). 14687696 10.1016/j.regpep.2003.09.004

[144] Price, C. J., Hoyda, T. D. & Ferguson, A. V. The area postrema: a brain monitor and integrator of systemic autonomic state. *Neuroscientist* **14**, 182–194 (2008). 18079557 10.1177/1073858407311100

[145] Karim, M. A., Leong, S. K. & Perwaiz, S. A. On the anatomical organization of the vagal nuclei. *Am. J. Primatol.* **1**, 277–292 (1981). 31995920 10.1002/ajp.1350010305

[146] Kalia, M. & Mesulam, M. M. Brain stem projections of sensory and motor components of the vagus complex in the cat: II. Laryngeal, tracheobronchial, pulmonary, cardiac, and gastrointestinal branches. *J. Comp. Neurol.* **193**, 467–508 (1980). 7440778 10.1002/cne.901930211

[147] Zhu, J.-N. & Wang, J.-J. The cerebellum in feeding control: possible function and mechanism. *Cell Mol. Neurobiol.* **28**, 469–478 (2008). 18027085 10.1007/s10571-007-9236-z PMC11515829

[148] Zhu, J.-N., Yung, W.-H., Kwok-Chong Chow, B., Chan, Y.-S. & Wang, J.-J. The cerebellar-hypothalamic circuits: potential pathways underlying cerebellar involvement in somatic-visceral integration. *Brain Res. Rev.* **52**, 93–106 (2006). 16497381 10.1016/j.brainresrev.2006.01.003

[149] Yeo, B. T. et al. The organization of the human cerebral cortex estimated by intrinsic functional connectivity. *J. Neurophysiol.* **106**, 1125–1165 (2011). 21653723 10.1152/jn.00338.2011 PMC3174820

[150] Kong, R. Q. et al. A network correspondence toolbox for quantitative evaluation of novel neuroimaging results. *Nat. Commun.* **16**, 2930 (2025). 40133295 10.1038/s41467-025-58176-9 PMC11937327

[151] Xia, M., Wang, J. & He, Y. BrainNet viewer: a network visualization tool for human brain connectomics. *PLoS ONE* **8**, e68910 (2013). 23861951 10.1371/journal.pone.0068910 PMC3701683

[152] Power, J. D., Barnes, K. A., Snyder, A. Z., Schlaggar, B. L. & Petersen, S. E. Spurious but systematic correlations in functional connectivity MRI networks arise from subject motion. *Neuroimage* **59**, 2142–2154 (2012). 22019881 10.1016/j.neuroimage.2011.10.018 PMC3254728

[153] Iglesias, J. E. et al. A computational atlas of the hippocampal formation using ex vivo, ultra-high resolution MRI: application to adaptive segmentation of in vivo MRI. *Neuroimage* **115**, 117–137 (2015). 25936807 10.1016/j.neuroimage.2015.04.042 PMC4461537

[154] Cohen, A. L. et al. Defining functional areas in individual human brains using resting functional connectivity MRI. *Neuroimage* **41**, 45–57 (2008). 18367410 10.1016/j.neuroimage.2008.01.066 PMC2705206

