# Supplementary material for: Cortical and subcortical mapping of the human allostatic–interoceptive system using 7 Tesla fMRI
Source: Nat Neurosci. 2025 Oct 23;28(11):2380–91. doi: 10.1038/s41593-025-02087-x (PMC12586188; doi:10.1038/s41593-025-02087-x)
Supplement: Supplementary file 1 — Supplementary Tables 1–3, Figs. 1 and 2 and Note. [file 41593_2025_2087_MOESM1_ESM.pdf]

nature neuroscience

Supplementary information

https://doi.org/10.1038/s41593-025-02087-x

Cortical and subcortical mapping of the human allostatic–interoceptive system using 7 Tesla fMRI

In the format provided by the authors and unedited

Supplementary Table 1. Correspondence between functional connectivity observed in the current study and tract-tracing results in non-human animals, demonstrating anatomical connections between cortical and subcortical ROIs.

		**Cortical regions**				
		**sgACC (BA25)**	**pACC (BA24, 32)**	**aMCC (BA24)**	**mvaIns/lvaIns**	**dmIns/dpIns**
**Cortical regions**	**sgACC (BA25)**					
	**pACC (BA24, 32)**	*†Fig. 1* (1)<br>†Fig. 5 (2)<br>†Fig. 2A (1)				
	**aMCC (BA24)**	*†Fig. 4* (3)<br>†Fig. 7 (2)<br>†Fig. 3A (1)	*†Fig. 5* (2)<br>*†Fig. 2A* (1)<br>†Fig. 7 (2)<br>†Fig. 3A (1)			
	**mvaIns/lvaIns**	*†Fig. 8* (4)<br>†Case M707 (5)	*†Fig. 5* (2)<br>†Case M776 (5)	*†Fig. 6* (2)<br>*†Fig. 1* (6)<br>†Fig. 1 (7)		
	**dmIns/dpIns**	*†Not observed* (1, 6, 8–10)<br>†Not observed (5, 6, 8, 9)	*†Fig. 5* (2)<br>†Not observed (5, 6, 8, 9)	*†Fig. 3* (6)<br>†Fig. 4 (7)	*†Fig. 1* (7)<br>†Fig. 4 (7)<br>†Fig. 1 (6)	

Note: Gray shading indicates connections that showed significant positive functional connectivity in the current study. Text describes the results of tract-tracing studies of non-human animals. Efferent connections (i.e., from the ROI in the column to the ROI in the row) are indicated in regular font, afferent connections (i.e., from the ROI in the row to the ROI in the column) are indicated in italic font. We considered two regions to share anatomical connections if the anatomical tracer injected in one region showed any terminations in the second region. * rat study, † monkey study, ʃ mouse study, ˆ cat study, " hamster study, ** rabbit study. Abbreviations: aMCC: anterior midcingulate cortex; dmIns: dorsal mid insula; dpIns: dorsal posterior insula; lvaIns: lateral ventral anterior insula; mvaIns: medial ventral anterior insula; pACC: pregenual anterior cingulate cortex; sgACC: subgenual anterior cingulate cortex.

Supplementary Table 1 (continued). Correspondence between functional connectivity observed in the current study and tract-tracing results in non-human animals, demonstrating anatomical connections between cortical and subcortical ROIs.

		**Cortical regions**				
		**sgACC (BA25)**	**pACC (BA24, 32)**	**aMCC (BA24)**	**mvaIns/lvaIns**	**dmIns/dpIns**
**Subcortical regions**	**mdThal**	^Fig 3 (11)<br>†Fig 4 (12)	^Fig 3 (11)<br>_†Fig 19(13)_	Not evident	_*Fig 6 (14)_<br>*Case R579 (15)<br>*Fig 3, 4, Table 1 (16) (bidirectional)	_*Fig 6A(17)_
	**LGN**	Not evident	_"Fig 7 (18)_	Not evident	*Not observed (6)	*Not observed (6)
	**Hypothal**	†Fig 4,10 (19)<br>†Fig 8 (20)<br>†Fig 2 (12)	_*Fig 3 (21)_<br>†Fig 4,9,12 (19)<br>†Fig 9 (20)<br>^Fig 3 (11)	†Fig 4 (19)	*Fig 3,5 (16)<br>*Fig 2 (21)<br>†Fig 3,6 (19) (bidirectional)	*Fig 2 (21)<br>†Fig 6 (19)
	**Hippo**	_†Fig 6 (22)_<br>_†Fig 3 (23)_	_†Fig 4 (22)_	_†Fig 5,7 (22)_<br>_†pg 440 (23)_	_*Fig 12 (24)_	_*Not observed (24)_
	**Amygdala**	†Fig 2,5,6 (25) (bidirectional)<br>†Fig 3,4 (26)<br>†Fig 2,5,6 (27)	^Fig 3 (11)<br>†Fig 2,6 (25) (bidirectional)<br>†Fig 3,4 (26)<br>†Fig 2 (27)	†Fig 2,5,6 (25) (bidirectional)<br>†Fig 3,4,5 (26)<br>†Fig 2,7 (27)	†Fig 2 (28)<br>†Fig 2 (29)<br>†Fig 3,4 (26)<br>†Fig 2 (27)	†Fig 3 (28)<br>†Fig 2,3,6 (29)<br>†Fig 3,4,5 (26)<br>†Fig 2,8,9 (27)
	**Striatum**	^Fig 3 (11)<br>†Fig 3-5 (30) (undirectional)	^Fig 3 (11)<br>^Fig 3 (11)<br>†Fig 3-5 (30) (undirectional)	†Fig 3-5 (30) (undirectional)	†Fig 3-15 (31)<br>†Fig 4,6,7,9 (32)	†Fig 3-15 (31)<br>†Fig 4,6-9,11 (32)
	**PAG**	†Fig 2-4 (12)<br>†Fig 1-6 (33)	^Fig 3 (11)<br>†Fig 1-6 (33)	†Fig 1-7 (33)	†Fig 1,7 (33)<br>*Fig 3,5 (16) (bidirectional)	†Fig 7 (33)<br>*Fig 7 (16)
	**DR**	†Fig 8 (12)	†Fig 12 (34)<br>*Fig 8 (35)	†Fig 12 (34)	*Fig 2 (35)<br>*Fig3,5 (16) (bidirectional)	*Not observed (35)
	**SC**	*Fig 15 (36)	*Fig 15 (36)	*Fig 15 (36)	*Fig 5 (37)	Not evident
	**SN**	*pg 1757 (36)	*pg 1757 (36)	*Not observed (36)	*Fig 3 (16)	Not evident
	**VTA**	*pg 1757 (36)	*pg 1757 (36)	*Not observed (36)	*Fig 3 (16)	Not evident
	**PBN**	†Fig 8 (12)	†Fig 12 (34)<br>^Fig 3 (11)	†Fig 12 (34)	*Fig 1,3 (38) (bidirectional)<br>*Fig 3,5[10] (bidirectional)	_*Fig 4 (14)_<br>*Fig 1,3 (38) (bidirectional)
	**LC**	Not evident	†Fig 12 (34)<br>_*Table 1 (39)_	†Fig 12 (34)	*Fig 5 (16)	Not evident
	**NTS**	†Not observed (12)<br>*Not observed (40)	*Not observed (40)<br>*Fig 1 (41)	*Not observed (40)	*Fig 4 (40)	*Fig 5 (42)

Note: Gray shading indicates connections that showed significant positive functional connectivity in the current study. Text describes the results of tract-tracing studies of non-human animals. Efferent connections (i.e., from cortical to subcortical) are indicated in regular font, afferent connections (i.e., from subcortical to cortical) are indicated in italic font. We considered two regions to share anatomical connections if the anatomical tracer injected in one region showed any terminations in the second region. We reviewed the entire striatum because many source studies did not differentiate dorsal vs. ventral portions of the striatum. * rat study, † monkey study, ʃ mouse study, ˆ cat study, " hamster study, ** rabbit study. Abbreviations: aMCC: anterior midcingulate cortex; dmIns: dorsal mid insula; dpIns: dorsal posterior insula; DR: dorsal raphe; Hippo: hippocampus; Hypothal: hypothalamus; LC: locus coeruleus; LGN: lateral geniculate nucleus; lvaIns: lateral ventral anterior insula; mvaIns: medial ventral anterior insula; mdThal: mediodorsal thalamus; NTS: nucleus tractus solitarius; pACC: pregenual anterior cingulate cortex; PAG: periaqueductal gray; PBN: parabrachial nucleus; SC: superior colliculus; sgACC: subgenual anterior cingulate cortex, SN: substantia nigra; VTA: ventral tegmental area.

Supplementary Table 1 (continued). Correspondence between functional connectivity observed in the current study and tract-tracing results in non-human animals, demonstrating anatomical connections between cortical and subcortical ROIs.

		**Subcortical regions**													
		**mdThal**	**LGN**	**Hypothal**	**Hippo**	**Amygdala**	**Striatum**	**PAG**	**DR**	**SC**	**SN**	**VTA**	**PBN**	**LC**	**NTS**
**Subcortical regions**	**mdThal**														
	**LGN**	Not evident													
	**Hypothal**	_*Fig 24 (43)_	*Fig 2 (44)<br>*Fig 9 (45)<br>*Fig 3 (46)												
	**Hippo**	Not evident	*Table 6 (47)	_*Fig 1 (48)_<br>_*Fig 3 (47)_											
	**Amygdala**	_*Fig 24 (43)_<br>_*Fig 5 (49)_	*Fig 3 (48)	_*Fig 1(50)_<br>_*Fig 1 (51)_	*Fig 5 (52)										
	**Striatum**	_*Fig 22 (43)_	Not evident	_*Fig 2,3_ (53)<br>_*Fig 1_ (15)	†Fig 5 (54)	†Fig 4 (54)<br>*Fig 2,3 (53)									
	**PAG**	_†Case SM-7,8_ (55)<br>*Fig 2 (56)<br>_*Fig 6_ (57)	_*Case R-43, 45, 59_ (58)	†Case SM-8 (55)<br>*Fig 9, 10 (57)<br>_*Case R-43, 45, 50, 59_ (58)	*Case 33 (59)	_*Case R-50,R-59_ (58)<br>^Case H3, 7, 19 (60)	Not evident								
	**DR**	_*Fig 2_ (61)	_*Fig 3_ (62)<br>^_Fig 1_(63)<br>_*Fig 3_ (64)	_*Fig 3_ (65)<br>_*Fig 9_ (61)	_*Fig 2_ (66)	_*Fig 2_ (61)	_*Fig 9, 13_ (61)	_*Case R-49, 50, 59_ (58)							
	**SC**	*Fig 10 _(43)_<br>*Not observed (67)	*Fig 3 (64)<br>_† Fig 1_(68)<br>*Fig 3 (69)<br>_*R27_ (58)	^_Fig 3_ (70)<br>**_Fig 1_ (71)	*Not observed (72)	_*Fig 4,6_ (73)<br>_*pg 403_ (74)	Not evident	^Fig 1(75) (bidirectional)<br>*Fig 2 (76)	*Table 2 (77)<br>*Fig 1 (62)						
	**SN**	_*Fig 26 (43)_<br>^Fig 4 (78)	^_Fig 1_ (63)	_*Fig 6_ (79)	_*pg 9_ (80)	_*Fig 1_(81)<br>_*Fig 1_ (82)<br>_*Fig 6_ (79)	_*Fig 2_ (83)<br>^_Fig 4_ (84)<br>_*Fig 3_ (85)<br>_*Fig 6_ (79)	_*Fig 2_ (86)<br>**Fig 4 (87)	*Fig 3 (88)<br>*Fig 2 (61)	^Fig 2 (89)<br>^Fig 2 (60)<br>*Fig 3 (90)					
	**VTA**	_*Fig 31(43)_<br>^Fig 3 (78)	Not evident	_*Fig 4_ (72)	*Not observed (72)	_*Fig 12_ (72)	_*Fig 12_ (72)	_*Fig 3_ (86)<br>**Fig 4 (87)	*Fig 2 (61)<br>*Fig 6 (65)	^_Fig 6_ (91)<br>*Fig 1 (90)<br>^Fig 1 (89)	_*Fig 12_ (72)				
	**PBN**	_*Fig 9R (43)_	^_Fig 1_ (92)<br>^Fig 1 (63)	"_Fig 3_ (93)<br>_*Fig 2_ (94)	_*Fig 2_ (95)	"_Fig 2_ (93)<br>_*Fig 1_ (96)	_*Fig 2_ (97)	_*Case R-43, 45, 50, 59_ (58)	_*Fig 6_ (65)	_*Fig 3_ (69)<br>_*Fig 3_ (98)	_*Fig 8_ (83)<br>*Fig 4 (79)	*Fig 2 (99)<br>*Fig 18 (72)			
	**LC**	_*Fig 9R (43)_<br>^Fig 5 (78)	_*Fig 8_ (100)<br>_*Fig 10_ (64)	*Table 1 (101)<br>*Fig 23 (102)<br>_*Fig 3_ (103)	_*Fig 1C_ (104)<br>_*Fig 2_ (95)<br>_*Fig 2_ (105)<br>_*Fig 3_ (103)	*Table 1C (101)	Not evident	_*Case R-43, 45, 50_ (58)	*Fig 15 (102)<br>_*Fig 3_ (106)<br>_(bidirectional)_	_*Fig 4,5_ (77)	*Not observed (79)	*Fig 1 (107)	*Fig 17 (102)		
	**NTS**	_*Fig 31 (43)_	Not evident	_*Fig 2_ (108)<br>ʃFig 1 (109)	*Not observed (95)	_*Fig 4_ (108)<br>_*Fig 5_ (110)	*Fig 1 (111)<br>*Fig 1 (112)<br>*Fig 4 (113)	_*Fig 3_ (86)<br>**Fig 4 (87)	_*Fig 2_ (114)	_*Fig 4_ (40)<br>*Not observed (113)	Not evident	_*Fig 2_ (115)<br>_*Table 2_ (116)<br>*Fig 1 (111)	"Fig 3 (93)<br>*Fig 1(113)<br>ʃFig 1 (109)	_*Fig 1_ (117)<br>_*Not observed_ (102)	

Note: Gray shading indicates connections that showed significant positive functional connectivity in the current study. Text describes the results of tract-tracing studies of non-human animals. Efferent connections (i.e., from the ROI in the column to the ROI in the row) are indicated in regular font, afferent connections (i.e., from the ROI in the row to the ROI in the column) are indicated in italic font. We considered two regions to share anatomical connections if the anatomical tracer injected in one region showed any terminations in the second region. We reviewed the entire striatum because many source studies did not differentiate dorsal vs. ventral portions of the striatum. * rat study, † monkey study, ʃ mouse study, ^ cat study, "hamster study, ** rabbit study. Abbreviations: DR: dorsal raphe; Hippo: hippocampus; Hypothal: hypothalamus; LC: locus coeruleus; LGN: lateral geniculate nucleus; mdThal: mediodorsal thalamus; NTS: nucleus tractus solitarius; PAG: periaqueductal gray; PBN: parabrachial nucleus; SC: superior colliculus; SN: substantia nigra; VTA: ventral tegmental area.

Supplementary Table 2. Functional connectivity of the superior parietal lobule (averaged Fisher's transformed *z*-scores). The seed coordinates of the SPL are MNI X, Y, Z = 46, 12, 29 (118).

ROI	Functional connectivity
mdThal	
LGN	0.02
Hypothal	
Hippo	0.12*
dAmy	2.50*
NAcc	
PAG	
DR	
SC	0.09*
SN	0.02
VTA	
PBN	
LC	
VSM	0.03*

Note. Empty cells indicate negative connectivity. *$p < 0.05$, two-tailed t-test. Abbreviations: dAmy: dorsal amygdala; DR: dorsal raphe; Hippo: hippocampus; Hypothal: hypothalamus; LC: locus coeruleus; LGN: lateral geniculate nucleus; mdThal: mediodorsal thalamus; NAcc: nucleus accumbens; PAG: periaqueductal gray; PBN: parabrachial nucleus; SC: superior colliculus; SN: substantia nigra; VSM: medullary viscero-sensory-motor nuclei complex, including the nucleus tractus solitarius, dorsal motor nucleus of the vagus, nucleus ambiguus, and hypoglossal nucleus; VTA: ventral tegmental area.

Supplementary Table 3. Percentage of subcortical ROI that overlapped with the subcortical connecting regions. Subcortical connecting regions were computed by taking the conjunction between the binarized maps ($p < 0.05$, two-tailed t-test) of all subcortically seeded functional connectivity maps ($N = 14$).

ROI	Percentage
mdThal	1.1
LGN	26.8
Hypothal	0.0
Hippo	1.8
dAmy	4.2
NAcc	0.2
PAG	12.0
DR	14.4
SC	11.4
SN	12.5
VTA	9.1
PBN	0.0
LC	0.0
VSM	0.0

Abbreviations: dAmy: dorsal amygdala; DR: dorsal raphe; Hippo: hippocampus; Hypothal: hypothalamus; LC: locus coeruleus; LGN: lateral geniculate nucleus; mdThal: mediodorsal thalamus; NAcc: nucleus accumbens; PAG: periaqueductal gray; PBN: parabrachial nucleus; SC: superior colliculus; SN: substantia nigra; VSM: medullary viscero-sensory-motor nuclei complex, including the nucleus tractus solitarius, dorsal motor nucleus of the vagus, nucleus ambiguus, and hypoglossal nucleus; VTA: ventral tegmental area.

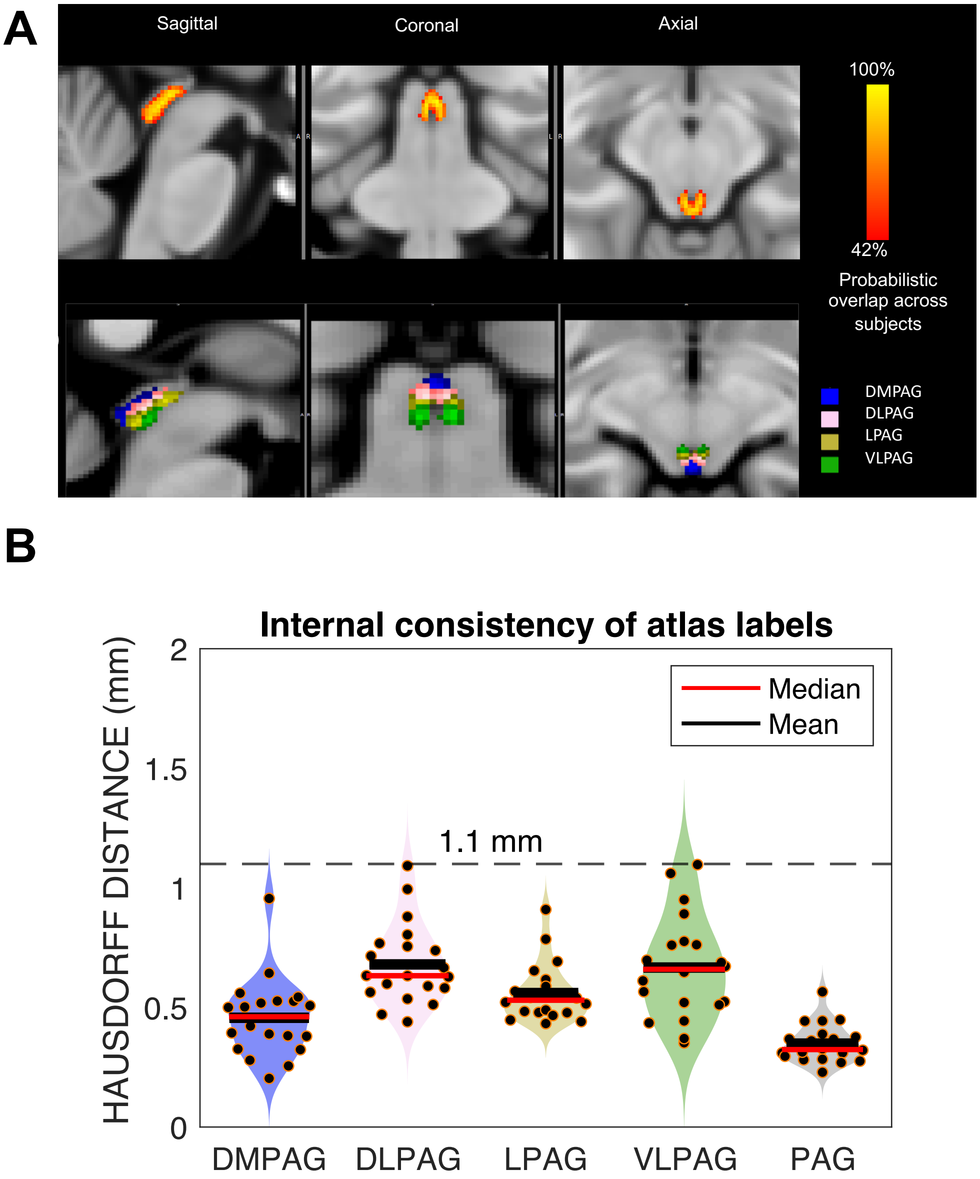

Supplementary Figure 1. (A) Probabilistic atlas label of PAG and dorsomedial, dorsolateral, lateral and ventrolateral PAG subregions (DMPAG, DLPAG, LPAG, VLPAG) based on a subset of 20 subjects. **(B)** Internal consistency of atlas label, which was below the 1.1 mm imaging resolution for each label (two-tailed t-test, $p < 0.05$). Abbreviations: DLPAG: dorsolateral periaqueductal gray; DMPAG: dorsomedial periaqueductal gray; LPAG: lateral periaqueductal gray; PAG: periaqueductal gray; VLPAG: ventrolateral periaqueductal gray.

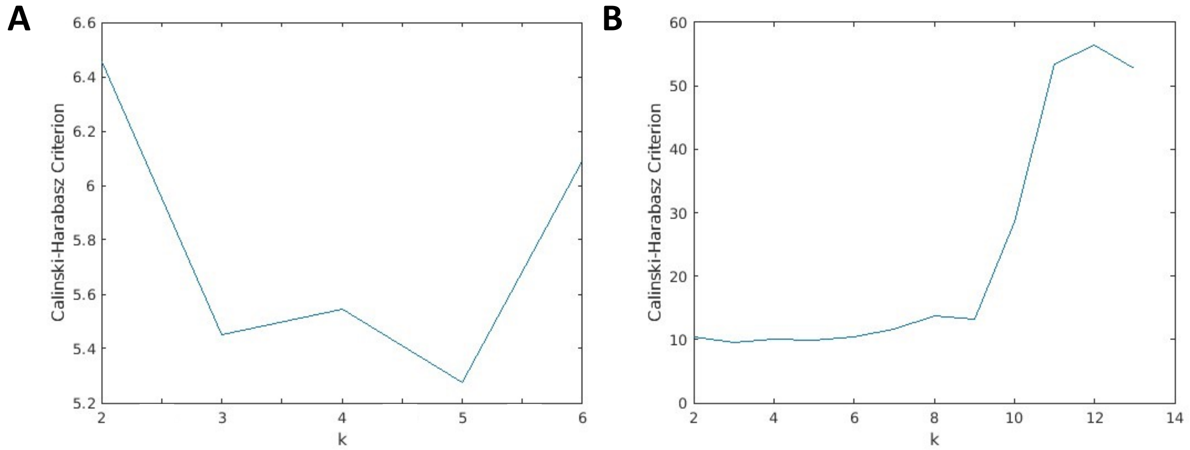

Supplementary Figure 2. Evaluation of optimal number of clusters for k-means. We calculated the Calinski-Harabasz Criterion (119) for a range of values of *k* using similarity matrices η^2 (120) based on cortical maps of **(A)** cortical seeds (for *k* = 2 to 6) and **(B)** subcortical seeds (for *k* = 2 to 10). Higher Calinski-Harabasz Criterion indicates larger between-cluster variance and smaller within-cluster variance, i.e., better solution.

Supplementary Notes

MRI acquisition. MRI data were acquired using a 7 Tesla scanner (Magnetom, Siemens Healthineers, Erlangen, Germany) with a 32-channel phased-array head coil. Participants completed a structural scan, three resting state scans, three diffusion-weighted scans, as well as other tasks unrelated to the current analysis. The structural scan was acquired using a high-resolution multi-echo echo-planar image (EPI, (121)) with the following parameters: repetition time (TR) = 8520 ms, echo time (TE) = 22 ms, flip angle (FA) = 90°, voxel size = 1.1 mm isotropic, in plane field of view (FOV) = 205 mm x 205 mm, bandwidth = 1414 Hz/pixel, echo spacing = 0.82 ms, 18 inversion times, GRAPPA-factor = 3. The resting state scans were acquired using a fast low-angle excitation echo-planar technique (122) using the following parameters: TR = 2340 ms, TE = 28 ms, FA = 75°, voxel size = 1.1 mm isotropic, FOV = 205 mm x 205 mm x 135.3 mm, nominal echo-spacing = 0.82 ms, readout bandwidth = 1414 Hz/pixel, N. slices =123, slice acquisition order = interleaved, N. repetitions = 256, phase encoding direction = anterior to posterior, acquisition time = 10'37" per scan, three scans. The diffusion-weighted scans were acquired using a spin-echo echo-planar sequence with parameters: TR = 5800 ms, TE = 63.2 ms, voxel size = 1.1 mm isotropic, N. slices = 61, slice orientation = transversal, readout bandwidth = 1414 Hz/pixel, nominal echo-spacing = 0.82 ms, FOV = 205 mm x 205 mm x 67.1 mm, phase encoding direction = anterior to posterior, GRAPPA-factor = 3, partial Fourier: 6/8, unipolar diffusion-weighting gradients, number of diffusion directions = 60 (b-value ~ 1000 s/mm^2), 7 interspersed "b0" images (non-diffusion weighted, b-value ~ 0 s/mm^2, also used as T_2-weighted MRI), 3 repetitions, acquisition time per repetition 6'58". Seven b0 images were also acquired with the phase encoding direction = posterior to anterior (acquisition time = 1'10").

Preprocessing of structural data. We used FSL (https://fsl.fmrib.ox.ac.uk/fsl/fslwiki), AFNI (https://afni.nimh.nih.gov/), Freesurfer (http://surfer.nmr.mgh.harvard.edu) and SPM (https://www.fil.ion.ucl.ac.uk/spm/) to preprocess the structural data. For each subject, the high-resolution structural EPI was first converted to a magnetization-prepared gradient-echo-like (MPRAGE-like) contrast (121), then reoriented to a standard orientation (FSL, reorient2std) and bias field corrected (SPM8, London, UK). This T_1-weighted EPI (T_1wEPI) approach has been shown to improve coregistration between structural and functional images, particularly in regions vulnerable to distortion due to magnetic susceptibility differences (121). We then ran standard surface reconstruction and parcellation, as well as subcortical segmentation using Freesurfer.

Preprocessing of fMRI data. The preprocessing pipeline for the resting state fMRI scans began with reorientation (FSL, fslreorient2std), slice timing correction (FSL, slicetimer), concatenation of all three resting state runs, coregistration to the structural T1wEPI (FSL, epi_reg using boundary-based registration), and motion correction (Freesurfer, preproc-sess -per-session). We then conducted nuisance regression (custom Matlab script performing general linear model fitting and regression out of covariates of no interest) to remove physiological noise due to motion, as well as due to non-BOLD and pulsatility effects evaluated in the white matter, ventricular cerebrospinal fluid, and the cerebral aqueduct. For the former, we used as nuisance regressors 6 motion parameters (rotations and translations) computed during motion correction. For the latter, we computed the mean time courses (FSL, fslmeants) across voxels of a white matter mask and six cerebrospinal fluid (CSF) masks, which contained voxels in both CSF-spaces distal to the brainstem (such as the lateral ventricle, inferior lateral ventricle, choroid plexus, third ventricle) and neighboring the brainstem (such as the fourth ventricle, and cerebral aqueduct). The cerebral aqueduct mask was manually defined for each subject by computing the signal standard deviation of the resting state time series and selecting the five voxels with the highest standard deviation. Previous work (123, 124) has shown that removal of the signal of the cerebral aqueduct and fourth ventricle is crucial for the functional connectivity analysis of adjacent brainstem nuclei, such as the periaqueductal gray and dorsal raphe. We then conducted temporal filtering (0.01-Hz high-pass filter and 0.08-Hz low-pass filter, AFNI, 3dFourier), and normalization of the fMRI from T1wEPI space to MNI152 space. The

latter was performed by computing the affine and non-linear transformation that aligned the T_1wEPI image to the 1mm-isotropic resolution T_1-weighted MNI152 template (Advanced Normalization Tool, ANTs, Philadelphia, USA), using parameters as in (123). The generic affine transformation was computed by concatenating center-of mass alignment, rigid, similarity and fully affine transformations. The high-dimensional non-linear transformation was a symmetric diffeomorphic normalization transformation with neighborhood cross correlation, regular sampling, gradient step size: 0.15, four multi-resolution levels, smoothing sigmas: 3, 2, 1, 0 voxels – fixed image space –, shrink factors: 6, 4, 2, 1 voxels – fixed image space –, histogram matching of images before registration, data winsorization – quantiles: 0.001, 0.999 –, convergence criterion: slope of the normalized energy profile over the last 10 iterations < 10^{-8}. The affine and non-linear transformations were then combined into a single warp field and were applied to the fMRI in T1wEPI space. For each dataset the quality of the coregistration was verified. Finally, we performed conversion to Freesurfer orientation/dimensions (FSL, fslswapdim, fslroi), detrending (FSL, fslmaths), minimal spatial smoothing (1.25mm full-width at half-maximum) (FSL, fslmaths), and resampling to cortical surfaces (Freesurfer, mri_vol2surf).

Seed definitions. Seven cortical (sgACC, pACC, aMCC, mvAIns, lvAIns, dmIns and dpIns) and dAmy seeds were defined using the procedure outlined in (118). Cortical seeds were first created using 4mm-radius spheres centered on the MNI coordinates that showed increased activity in previous task-dependent fMRI studies of interoception: dorsal mid insula (dmIns) – 41, 2, 3 (125); dorsal posterior insula (dpIns) – 36, -32, 16 (126); medial ventral anterior insula (mvAIns) – 30, 16, -14 (127); lateral ventral anterior insula (lvAIns) – 44, 6, -15 (128); pregenual anterior cingulate cortex (pACC) – 13, 44, 0 (126); anterior mid cingulate cortex (aMCC) – 9, 22, 33 (128); subgenual anterior cingulate cortex (sgACC) – 2, 14, -6 (129). We found the vertex on the MNI152 pial surface that is closest to each individual subject's cortex and smoothed it by 4 mm. The individual cortical label was projected back into the subject's native volumetric space to calculate the averaged time series within the seed. We directly projected a spherical dorsal amygdala (dAmy) seed – centered on MNI 27, 3, -12 (130) – into each subject's native volumetric space and calculated the averaged time series within the seed. PAG and its four subregions (dorsomedial, dorsolateral, lateral, ventrolateral), were first manually defined in 20 individual subjects based on their diffusion-weighted scans and then the group probabilistic map was thresholded at 35% to generate a group label (**Supplementary Figure 1**; also see section below on *Delineation of the periaqueductal gray and its subregions*). Brainstem nuclei were delineated semi-automatically via 7 Tesla multi-contrast (diffusion and T2-weighted) MRI using neighboring landmarks. Specifically, seven brainstem nuclei seeds were based on the binary labels (35%) from the Brainstem Navigator toolkit (https://www.nitrc.org/projects/brainstemnavig/). Specific Brainstem Navigator labels used for each seed are listed in parentheses following the seed name): 1) locus coeruleus (LC_l, LC_r) (131), 2) lateral geniculate nucleus (LG_r, LG_l) (132), 3) parabrachial nucleus (LPB_l, LPB_r, MPB_l, MPB_r) (133), 4) medullary viscero-sensory-motor nuclei complex (which includes the nucleus tractus solitarius, as well as the vagus nerve nucleus, hypoglossal nucleus, prepositus, intercalated nucleus, and interpositus nucleus; VSM_l, VSM_r) (133), 5) dorsal raphe (updated label, see SI; DR) (123), 6) substantia nigra (SN_l, SN_r) (123), and 7) ventral tegmental area (VTA_PBP_l, VTA_PBP_r) (134). The 8th brainstem nucleus, the superior colliculus, and its subregions of superficial and deep layers were defined using a hand-drawn mask template, which was semi-automatically refined based on neighboring landmarks for each subject (135, 136). The medial dorsal thalamus seed was from the medial dorsal thalamus nucleus, defined based on (137), compiled in CANLAB Combined Atlas 2018 (https://github.com/canlab/Neuroimaging_Pattern_Masks/tree/master/Atlases_and_parcellations/2018_Wager_combined_atlas). Hippocampal subregions of head, body, and tail were derived using the longitudinal segmentation method as implemented in Freesurfer (138). The hypothalamic (139) and nucleus accumbens seeds were directly generated from Freesurfer subcortical segmentation (140). Subregions of the hypothalamus were functionally parcellated using Louvain community detection algorithm (141).

Preprocessing of diffusion weighted images. In a subset of 20 subjects, diffusion weighted images were concatenated across three scans, rotated to standard orientation (FSL, reorient2std), motion- and distortion-corrected (FSL) as in (142). We then computed the diffusion tensor invariants (e.g., fractional anisotropy, FA, mean diffusivity, MD) and S_0 image (employed as T_2-weighted MRI) using FSL (dtifit). After reorienting the S_0 image to a standard orientation (FSL, reorient2std), and performing bias field correction (SPM8, London, UK), we computed the affine transformation that aligned it to the T_1wEPI image. For each subject, the FA map, S_0 image and PAG delineation (see below) were then coregistered to MNI space by concatenating the affine transformation mapping the S_0 image to the T_1wEPI, with the affine and the non-linear transformations (described above) mapping the T_1wEPI to the 1mm-isotropic resolution T_1-weighted MNI152.

Delineation of the periaqueductal gray and its subregions. In each of 20 subjects, an expert rater (M.B.) manually delineated the label (i.e., binary mask) of a cylindrical region surrounding the cerebral aqueduct (defined from the mean diffusivity image, as MD > 0.0015) and hyperintense in the S_0 image (T_2-weighted MRI), which comprised both the periaqueductal gray (PAG) and the upper part of the dorsal raphe (DR). Then based on the FA contrast, the upper DR was manually discriminated from the PAG (the DR was darker than the ventrolateral part of the PAG), and two labels (PAG, upper DR) were generated for each subject. Then, within the whole PAG label, M.B. manually delineated four bilateral PAG subregions (dorsomedial, DMPAG, dorsolateral, DLPAG, lateral, LPAG, and ventrolateral, VLPAG) in native space using a postmortem brainstem atlas (143) as a guide to define the neighborhood relationships and location of PAG subregions within the PAG. The DMPAG was defined as the column hypointense in FA posterior to the aqueduct; the DLPAG was defined as a roughly triangular prism hypointense in FA, anterolateral to DMPAG and extending only in the upper part of the PAG; LPAG was identified as a roughly triangular prism hypointense in FA, lateral to the aqueduct and anterolateral to DLPAG; finally, VLPAG was defined as a roughly triangular prism slightly hyperintense compared to the other subregions, anterolateral to LPAG and extending only in the lower part of the PAG. Single-subject labels of the whole PAG and PAG subregions were coregistered to MNI space as explained above. Then, we computed the spatial overlap (range: 0-100%) of these labels across subjects to yield probabilistic atlas labels of the PAG and its subregions in MNI space. Each probabilistic atlas label was validated by computing its *internal consistency* across subjects, as the modified Hausdorff distance between each label and the probabilistic atlas label (thresholded at 35%) generated by averaging the labels across the other subjects (leave-one-out cross validation). Note that due to the limited coverage of the diffusion weighted imaging protocol only the upper part of the DR was covered in this study; thus, DR delineations were not used to generate a DR probabilistic atlas label. Rather, M.B. manually improved the semi-automatic delineations of DR of (142) to ensure contiguity between them and the newly generated PAG probabilistic label, which yielded an updated DR probabilistic label.

Functional connectivity analysis. To avoid Type II error from stringent group-level threshold that decrease the ability of detecting small, but reliable connectivity signals (144), we opted to separate signal from noise using a bootstrapping analysis. We randomly resampled 80% of the sample ($N = 72$) 1000 times. In each iteration, we estimated cortical connectivity using a combination of surface- and volume-based analyses, as outlined in (118). Specifically, on the subject level, we ran a voxel-wise regression on left and right hemispheres of MNI152 and subcortical volume of MNI305 to compute the contrast effect size (contrast * beta) of the seed time series. On the group level, we concatenated the contrast effect size maps from all subjects and ran a general linear model analysis to test whether the group mean differed from zero (two-tailed one-sample t-test). This yielded final group maps that showed regions whose fluctuations significantly correlated with the seed's BOLD time series. We binarized the group maps to retain positive connectivity surviving the liberal threshold of $p < .05$ and summed the binarized maps across all 1000 iterations to obtain a 'bootstrapped' connectivity map that ranges between 0-1000.

Evaluating the optimal number of clusters (k) for k-means clustering analyses. We ran k-means clustering analyses with a range of *k values* (k = 2 to 6 for seven cortical seeds and k = 2 to 13 for 14 subcortical seeds). For both sets of analyses, we used 10 initializations of new centroid positions with a maximum of 1000 iterations each to find the lowest local minimum for sum of distances (*kmeans,* MATLAB). We calculated the Calinski-Harabasz Criterion (119) for each analysis (*evalclusters*, MATLAB). A higher Calinski-Harabasz Criterion indicates larger between-cluster variance and smaller within-cluster variance (i.e., a better clustering solution). Cortical maps derived from cortical seeds yielded in an unambiguously optimal solution with k = 2 based on the Calinski-Harabasz Criterion (**Supplementary Figure 2A**). For cortical maps derived from subcortical seeds, the Calinski-Harabasz Criterion remained stable for solutions between k = 2 to 9 but showed a drastic increase at k > 10 (**Supplementary Figure 2B**). To optimize parsimony (i.e., preferring a solution with fewer clusters when the evaluation criterion is similar) and consistency between analyses (i.e., aligning with the previous two-cluster solution for cortically seeded maps), we investigated the conjunction maps for k = 2 to 4 for interpretability.

Comparing k-means solutions for interpretability. The two-cluster solution differentiated the discovery maps with sparse cortical connectivity from those showing more widespread cortical connectivity (rather than differentiating spatially distinct connectivity patterns per se). The cluster with widespread connectivity included discovery maps from larger seeds in the mdThal, LGN, hippocampus, dAmy, NAcc, SC, SN and VTA and showed connectivity to large swaths of the cingulate (subgenual, pregenual, anterior mid, and posterior), insular (anterior to posterior), and frontal (medial prefrontal and superior frontal) cortices. It was observed in the three-cluster solution as well (Cluster 3; **Figure 5**). The sparse cluster from the two-cluster solution was further divided into two almost non-overlapping clusters in the three-cluster solution, one that included maps from seeds in the lower brainstem (LC, PBN, VSM), which primarily showed connectivity to the posterior cingulate cortex, supramarginal gyrus and some medial and lateral occipital regions (Cluster 1; **Figure 5**), and one that included maps from seeds in the upper brainstem (PAG, DR) and the hypothalamus, which showed connectivity to the aMCC and parahippocampal gyrus (Cluster 2; **Figure 5**). The four-cluster solution yielded the same clusters as the three-cluster solution, with the exception that the hippocampal seeded map split off from the dense cluster to form its own cluster and did not further improve solution interpretability.

Ruling out partial volume effects. Given the size of the brainstem seeds in relation to the voxel size, we sought to rule out partial volume effects. First, we eroded each brainstem seed by one voxel. For five seeds, eroding by one voxel reduced the seed to less than ten total voxels: VTA, DR, PBN, LC, and VSM. For these seeds, we dilated neighboring seeds by one voxel and subtracted those voxels from the target seed. Thus, an eroded VTA seed was created by subtracting dilated hypothalamus and SN seeds, an eroded DR seed was created by subtracting a dilated PAG seed, an eroded PBN seed was created by subtracting a dilated LC seed, and an eroded LC seed was created by subtracting a dilated PBN seed. VSM neighbored no other seeds. The time series of the eroded seeds were highly correlated with the time series of the full seeds (*M rs* = .74 - .99; *s.d. rs* = .007 - .11), suggesting that the signals from the outer most voxels of the seeds were not significantly impacted by potential noise introduced by partial volume effects. For this reason, we ruled out the possibility that partial volume effects contaminated functional connectivity between the brainstem seeds and the rest of the brain.

References

1. B. A. Vogt, D. N. Pandya, Cingulate cortex of the rhesus monkey: II. Cortical afferents. *J Comp Neurol* **262**, 271–289 (1987).
2. R. J. Morecraft, *et al.*, Cytoarchitecture and cortical connections of the anterior cingulate and adjacent somatomotor fields in the rhesus monkey. *Brain Res Bull* **87**, 457–497 (2012).
3. D. N. Pandya, G. W. Van Hoesen, M. M. Mesulam, Efferent connections of the cingulate gyrus in the rhesus monkey. *Exp Brain Res* **42**, 319–330 (1981).
4. S. T. Carmichael, J. L. Price, Connectional networks within the orbital and medial prefrontal cortex of macaque monkeys. *J Comp Neurol* **371**, 179–207 (1996).
5. T. Chiba, T. Kayahara, K. Nakano, Efferent projections of infralimbic and prelimbic areas of the medial prefrontal cortex in the Japanese monkey, Macaca fuscata. *Brain Res* **888**, 83–101 (2001).
6. M. M. Mesulam, E. J. Mufson, Insula of the old world monkey. III: Efferent cortical output and comments on function. *J Comp Neurol* **212**, 38–52 (1982).
7. E. J. Mufson, M.-M. Mesulam, Insula of the old world monkey. II: Afferent cortical input and comments on the claustrum. *J. Comp. Neurol.* **212**, 23–37 (1982).
8. R. J. Morecraft, G. W. Van Hoesen, Convergence of limbic input to the cingulate motor cortex in the rhesus monkey. *Brain Res Bull* **45**, 209–232 (1998).
9. K. S. Saleem, H. Kondo, J. L. Price, Complementary circuits connecting the orbital and medial prefrontal networks with the temporal, insular, and opercular cortex in the macaque monkey. *J Comp Neurol* **506**, 659–693 (2008).
10. H. Barbas, H. Ghashghaei, S. M. Dombrowski, N. L. Rempel-Clower, Medial prefrontal cortices are unified by common connections with superior temporal cortices and distinguished by input from memory-related areas in the rhesus monkey. *J Comp Neurol* **410**, 343–367 (1999).
11. P. Room, F. T. Russchen, H. J. Groenewegen, A. H. M. Lohman, Efferent connections of the prelimbic (area 32) and the infralimbic (area 25) cortices: An anterograde tracing study in the cat. *Journal of Comparative Neurology* **242**, 40–55 (1985).
12. L. J. Freedman, T. R. Insel, Y. Smith, Subcortical projections of area 25 (subgenual cortex) of the macaque monkey. *J Comp Neurol* **421**, 172–188 (2000).
13. J. P. Ray, J. L. Price, The organization of projections from the mediodorsal nucleus of the thalamus to orbital and medial prefrontal cortex in macaque monkeys. *Journal of Comparative Neurology* **337**, 1–31 (1993).
14. Z. H. Zhang, S. M. Oppenheimer, Baroreceptive and somatosensory convergent thalamic neurons project to the posterior insular cortex in the rat. *Brain Res* **861**, 241–256 (2000).
15. G. V. Allen, C. B. Saper, K. M. Hurley, D. F. Cechetto, Organization of visceral and limbic connections in the insular cortex of the rat. *J. Comp. Neurol.* **311**, 1–16 (1991).
16. L. Jasmin, A. Granato, P. T. Ohara, Rostral agranular insular cortex and pain areas of the central nervous system: A tract-tracing study in the rat. *Journal of Comparative Neurology* **468**, 425–440 (2004).
17. M. Nakashima, *et al.*, An anterograde and retrograde tract-tracing study on the projections from the thalamic gustatory area in the rat: distribution of neurons projecting to the insular cortex and amygdaloid complex. *Neuroscience Research* **36**, 297–309 (2000).
18. L. P. Morin, J. H. Blanchard, Forebrain connections of the hamster intergeniculate leaflet: comparison with those of ventral lateral geniculate nucleus and retina. *Vis Neurosci* **16**, 1037–1054 (1999).

19. D. Ongür, X. An, J. L. Price, Prefrontal cortical projections to the hypothalamus in macaque monkeys. *J Comp Neurol* **401**, 480–505 (1998).
20. N. L. Rempel-Clower, H. Barbas, Topographic organization of connections between the hypothalamus and prefrontal cortex in the rhesus monkey. *J Comp Neurol* **398**, 393–419 (1998).
21. S. Cavdar, *et al.*, The afferent connections of the posterior hypothalamic nucleus in the rat using horseradish peroxidase. *J Anat* **198**, 463–472 (2001).
22. H. Barbas, G. J. Blatt, Topographically specific hippocampal projections target functionally distinct prefrontal areas in the rhesus monkey. *Hippocampus* **5**, 511–533 (1995).
23. R. Insausti, M. Muñoz, Cortical projections of the non-entorhinal hippocampal formation in the cynomolgus monkey (Macaca fascicularis). *Eur J Neurosci* **14**, 435–451 (2001).
24. L. A. Cenquizca, L. W. Swanson, Spatial organization of direct hippocampal field CA1 axonal projections to the rest of the cerebral cortex. *Brain Res Rev* **56**, 1–26 (2007).
25. H. T. Ghashghaei, C. C. Hilgetag, H. Barbas, Sequence of information processing for emotions based on the anatomic dialogue between prefrontal cortex and amygdala. *Neuroimage* **34**, 905–923 (2007).
26. J. P. Aggleton, M. J. Burton, R. E. Passingham, Cortical and subcortical afferents to the amygdala of the rhesus monkey (Macaca mulatta). *Brain Res* **190**, 347–368 (1980).
27. L. Stefanacci, D. G. Amaral, Some observations on cortical inputs to the macaque monkey amygdala: an anterograde tracing study. *J Comp Neurol* **451**, 301–323 (2002).
28. E. J. Mufson, M. M. Mesulam, D. N. Pandya, Insular interconnections with the amygdala in the rhesus monkey. *Neuroscience* **6**, 1231–1248 (1981).
29. M. Höistad, H. Barbas, Sequence of information processing for emotions through pathways linking temporal and insular cortices with the amygdala. *Neuroimage* **40**, 1016–1033 (2008).
30. S. N. Haber, K.-S. Kim, P. Mailly, R. Calzavara, Reward-related cortical inputs define a large striatal region in primates that interface with associative cortical connections, providing a substrate for incentive-based learning. *J Neurosci* **26**, 8368–8376 (2006).
31. M. Chikama, N. R. McFarland, D. G. Amaral, S. N. Haber, Insular cortical projections to functional regions of the striatum correlate with cortical cytoarchitectonic organization in the primate. *J Neurosci* **17**, 9686–9705 (1997).
32. J. L. Fudge, M. A. Breitbart, M. Danish, V. Pannoni, Insular and Gustatory Inputs to the Caudal Ventral Striatum in Primates. *J Comp Neurol* **490**, 101–118 (2005).
33. X. An, R. Bandler, D. Ongür, J. L. Price, Prefrontal cortical projections to longitudinal columns in the midbrain periaqueductal gray in macaque monkeys. *J Comp Neurol* **401**, 455–479 (1998).
34. L. J. Porrino, P. S. Goldman-Rakic, Brainstem innervation of prefrontal and anterior cingulate cortex in the rhesus monkey revealed by retrograde transport of HRP. *J Comp Neurol* **205**, 63–76 (1982).
35. C. Peyron, J. M. Petit, C. Rampon, M. Jouvet, P. H. Luppi, Forebrain afferents to the rat dorsal raphe nucleus demonstrated by retrograde and anterograde tracing methods. *Neuroscience* **82**, 443–468 (1998).
36. C. Fillinger, I. Yalcin, M. Barrot, P. Veinante, Efferents of anterior cingulate areas 24a and 24b and midcingulate areas 24a′ and 24b′ in the mouse. *Brain Struct Funct* **223**, 1747–1778 (2018).

37. E. Comoli, *et al.*, Segregated Anatomical Input to Sub-Regions of the Rodent Superior Colliculus Associated with Approach and Defense. *Frontiers in Neuroanatomy* **6**, 9 (2012).
38. C. B. Saper, Reciprocal parabrachial-cortical connections in the rat. *Brain Res* **242**, 33–40 (1982).
39. D. M. Finch, E. L. Derian, T. L. Babb, Afferent fibers to rat cingulate cortex. *Experimental Neurology* **83**, 468–485 (1984).
40. D. van der Kooy, L. Y. Koda, J. F. McGinty, C. R. Gerfen, F. E. Bloom, The organization of projections from the cortex, amygdala, and hypothalamus to the nucleus of the solitary tract in rat. *J Comp Neurol* **224**, 1–24 (1984).
41. R. R. Terreberry, E. J. Neafsey, Rat medial frontal cortex: a visceral motor region with a direct projection to the solitary nucleus. *Brain Research* **278**, 245–249 (1983).
42. Y. Yasui, C. D. Breder, C. B. Saper, D. F. Cechetto, Autonomic responses and efferent pathways from the insular cortex in the rat. *J Comp Neurol* **303**, 355–374 (1991).
43. H. J. Groenewegen, Organization of the afferent connections of the mediodorsal thalamic nucleus in the rat, related to the mediodorsal-prefrontal topography. *Neuroscience* **24**, 379–431 (1988).
44. J. P. Card, R. Y. Moore, Organization of lateral geniculate-hypothalamic connections in the rat. *Journal of Comparative Neurology* **284**, 135–147 (1989).
45. R. Y. Moore, R. Weis, M. M. Moga, Efferent projections of the intergeniculate leaflet and the ventral lateral geniculate nucleus in the rat. *Journal of Comparative Neurology* **420**, 398–418 (2000).
46. J. D. Mikkelsen, A neuronal projection from the lateral geniculate nucleus to the lateral hypothalamus of the rat demonstrated with Phaseolus vulgaris leucoagglutinin tracing. *Neuroscience Letters* **116**, 58–63 (1990).
47. Atoji, Y., Wild, M., Fiber connections of the hippocampal formation and septum and subdivisions of the hippocampal formation in the pigeon as revealed by tract tracing and kainic acid lesions - Atoji - 2004 - Journal of Comparative Neurology - Wiley Online Library. Available at: https://onlinelibrary.wiley.com/doi/full/10.1002/cne.20186 [Accessed 17 December 2021].
48. N. S. Canteras, L. W. Swanson, Projections of the ventral subiculum to the amygdala, septum, and hypothalamus: A PHAL anterograde tract-tracing study in the rat. *Journal of Comparative Neurology* **324**, 180–194 (1992).
49. W. Young, G. Alheid, L. Heimer, The ventral pallidal projection to the mediodorsal thalamus: a study with fluorescent retrograde tracers and immunohistofluorescence. *J Neurosci* **4**, 1626–1638 (1984).
50. C. M. F. Prewitt, J. P. Herman, Anatomical interactions between the central amygdaloid nucleus and the hypothalamic paraventricular nucleus of the rat: a dual tract-tracing analysis. *Journal of Chemical Neuroanatomy* **15**, 173–186 (1998).
51. T. S. Gray, M. E. Carney, D. J. Magnuson, Direct projections from the central amygdaloid nucleus to the hypothalamic paraventricular nucleus: possible role in stress-induced adrenocorticotropin release. *Neuroendocrinology* **50**, 433–446 (1989).
52. R. C. Saunders, D. L. Rosene, G. W. V. Hoesen, Comparison of the efferents of the amygdala and the hippocampal formation in the rhesus monkey: II. Reciprocal and non-reciprocal connections. *Journal of Comparative Neurology* **271**, 185–207 (1988).

53. G. J. Kirouac, P. K. Ganguly, Topographical organization in the nucleus accumbens of afferents from the basolateral amygdala and efferents to the lateral hypothalamus. *Neuroscience* **67**, 625–630 (1995).
54. D. P. Friedman, J. P. Aggleton, R. C. Saunders, Comparison of hippocampal, amygdala, and perirhinal projections to the nucleus accumbens: Combined anterograde and retrograde tracing study in the Macaque brain. *Journal of Comparative Neurology* **450**, 345–365 (2002).
55. P. W. Mantyh, Forebrain projections to the periaqueductral gray in the monkey, with observations in the cat and rat. *Journal of Comparative Neurology* **206**, 146–158 (1982).
56. R. H. Thompson, L. W. Swanson, Organization of inputs to the dorsomedial nucleus of the hypothalamus: a reexamination with Fluorogold and PHAL in the rat. *Brain Res Brain Res Rev* **27**, 89–118 (1998).
57. E. Carstens, J. Leah, J. Lechner, M. Zimmermann, Demonstration of extensive brainstem projections to medial and lateral thalamus and hypothalamus in the rat. *Neuroscience* **35**, 609–626 (1990).
58. A. J. Beitz, The organization of afferent projections to the midbrain periaqueductal gray of the rat. *Neuroscience* **7**, 133–159 (1982).
59. J. M. Wyss, L. W. Swanson, W. M. Cowan, A study of subcortical afferents to the hippocampal formation in the rat. *Neuroscience* **4**, 463–476 (1979).
60. D. A. Hopkins, G. Holstege, Amygdaloid projections to the mesencephalon, pons and medulla oblongata in the cat. *Exp Brain Res* **32**, 529–547 (1978).
61. R. P. Vertes, A PHA-L analysis of ascending projections of the dorsal raphe nucleus in the rat. *Journal of Comparative Neurology* **313**, 643–668 (1991).
62. M. J. Villar, M. L. Vitale, T. Hökfelt, A. A. J. Verhofstad, Dorsal raphe serotoninergic branching neurons projecting both to the lateral geniculate body and superior colliculus: A combined retrograde tracing–immunohistochemical study in the rat. *Journal of Comparative Neurology* **277**, 126–140 (1988).
63. H. C. Hughes, W. H. Mullikin, Brainstem afferents to the lateral geniculate nucleus of the cat. *Exp Brain Res* **54**, 253–258 (1984).
64. D. A. Pasquier, M. J. Villar, Subcortical projections to the lateral geniculate body in the rat. *Exp Brain Res* **48**, 409–419 (1982).
65. P. Kalén, M. Karlson, L. Wiklund, Possible excitatory amino acid afferents to nucleus raphe dorsalis of the rat investigated with retrograde wheat germ agglutinin and D-[3H]aspartate tracing. *Brain Res* **360**, 285–297 (1985).
66. L. C. A. Conrad, C. M. Leonard, D. W. Pfaff, Connections of the median and dorsal raphe nuclei in the rat: An autoradiographic and degeneration study. *Journal of Comparative Neurology* **156**, 179–205 (1974).
67. K. E. Krout, A. D. Loewy, G. W. M. Westby, P. Redgrave, Superior colliculus projections to midline and intralaminar thalamic nuclei of the rat. *Journal of Comparative Neurology* **431**, 198–216 (2001).
68. J. K. Harting, M. F. Huerta, T. Hashikawa, D. P. van Lieshout, Projection of the mammalian superior colliculus upon the dorsal lateral geniculate nucleus: Organization of tectogeniculate pathways in nineteen species. *Journal of Comparative Neurology* **304**, 275–306 (1991).
69. S. B. Edwards, C. L. Ginsburgh, C. K. Henkel, B. E. Stein, Sources of subcortical projections to the superior colliculus in the cat. *J Comp Neurol* **184**, 309–329 (1979).

70. R. W. Rieck, M. F. Huerta, J. K. Harting, J. T. Weber, Hypothalamic and ventral thalamic projections to the superior colliculus in the cat. *J Comp Neurol* **243**, 249–265 (1986).
71. J. H. Fallon, R. Y. Moore, Superior colliculus efferents to the hypothalamus. *Neuroscience Letters* **14**, 265–270 (1979).
72. L. W. Swanson, The projections of the ventral tegmental area and adjacent regions: A combined fluorescent retrograde tracer and immunofluorescence study in the rat. *Brain Research Bulletin* **9**, 321–353 (1982).
73. R. Linke, A. d. De Lima, H. Schwegler, H.-C. Pape, Direct synaptic connections of axons from superior colliculus with identified thalamo-amygdaloid projection neurons in the rat: Possible substrates of a subcortical visual pathway to the amygdala. *Journal of Comparative Neurology* **403**, 158–170 (1999).
74. N. N. Doron, J. E. Ledoux, Organization of projections to the lateral amygdala from auditory and visual areas of the thalamus in the rat. *Journal of Comparative Neurology* **412**, 383–409 (1999).
75. M. Wiberg, Reciprocal connections between the periaqueductal gray matter and other somatosensory regions of the cat mid brain: A possible mechanism of pain inhibition. *Upsala Journal of Medical Sciences* **97**, 37–47 (1992).
76. N. S. Floyd, J. L. Price, A. T. Ferry, K. A. Keay, R. Bandler, Orbitomedial prefrontal cortical projections to distinct longitudinal columns of the periaqueductal gray in the rat. *Journal of Comparative Neurology* **422**, 556–578 (2000).
77. B. D. Waterhouse, B. Border, L. Wahl, G. A. Mihailoff, Topographic organization of rat locus coeruleus and dorsal raphe nuclei: Distribution of cells projecting to visual system structures. *Journal of Comparative Neurology* **336**, 345–361 (1993).
78. J. L. Velayos, F. Reinoso-Suarez, Topographic organization of the brainstem afferents to the mediodorsal thalamic nucleus. *J. Comp. Neurol.* **206**, 17–27 (1982).
79. R. M. Beckstead, V. B. Domesick, W. J. H. Nauta, Efferent connections of the substantia nigra and ventral tegmental area in the rat. *Brain Research* **175**, 191–217 (1979).
80. A. Gasbarri, A. Sulli, M. G. Packard, The dopaminergic mesencephalic projections to the hippocampal formation in the rat. *Progress in Neuro-Psychopharmacology and Biological Psychiatry* **21**, 1–22 (1997).
81. Y. Shinonaga, M. Takada, N. Mizuno, Direct projections from the central amygdaloid nucleus to the globus pallidus and substantia nigra in the cat. *Neuroscience* **51**, 691–703 (1992).
82. J. B. Rosen, J. M. Hitchcock, C. B. Sananes, M. J. D. Miserendino, M. Davis, A direct projection from the central nucleus of the amygdala to the acoustic startle pathway: Anterograde and retrograde tracing studies. *Behavioral Neuroscience* **105**, 817–825 (1991).
83. C. R. Gerfen, The neostriatal mosaic. I. compartmental organization of projections from the striatum to the substantia nigra in the rat. *Journal of Comparative Neurology* **236**, 454–476 (1985).
84. V. Anaya-Martinez, A. Martinez-Marcos, D. Martinez-Fong, J. Aceves, D. Erlij, Substantia nigra compacta neurons that innervate the reticular thalamic nucleus in the rat also project to striatum or globus pallidus: Implications for abnormal motor behavior. *Neuroscience* **143**, 477–486 (2006).
85. M. von Krosigk, Y. Smith, J. P. Bolam, A. D. Smith, Synaptic organization of gabaergic inputs from the striatum and the globus pallidus onto neurons in the substantia nigra and

retrorubral field which project to the medullary reticular formation. *Neuroscience* **50**, 531–549 (1992).
86. G. J. Kirouac, S. Li, G. Mabrouk, GABAergic projection from the ventral tegmental area and substantia nigra to the periaqueductal gray region and the dorsal raphe nucleus. *J Comp Neurol* **469**, 170–184 (2004).
87. S. T. Meller, B. J. Dennis, Efferent projections of the periaqueductal gray in the rabbit. *Neuroscience* **40**, 191–216 (1991).
88. D. Wirtshafter, T. R. Stratford, K. E. Asin, Evidence that serotonergic projections to the substantia nigra in the rat arise in the dorsal, but not the median, raphe nucleus. *Neuroscience Letters* **77**, 261–266 (1987).
89. J. G. McHaffie, *et al.*, A direct projection from superior colliculus to substantia nigra pars compacta in the cat. *Neuroscience* **138**, 221–234 (2006).
90. E. Comoli, *et al.*, A direct projection from superior colliculus to substantia nigra for detecting salient visual events. *Nat Neurosci* **6**, 974–980 (2003).
91. G. R. Leichnetz, *et al.*, Afferent and efferent connections of the cholinoceptive medial pontine reticular formation (region of the ventral tegmental nucleus) in the cat. *Brain Research Bulletin* **22**, 665–688 (1989).
92. D. J. Uhlrich, J. B. Cucchiaro, S. M. Sherman, The projection of individual axons from the parabrachial region of the brain stem to the dorsal lateral geniculate nucleus in the cat. *J. Neurosci.* **8**, 4565–4575 (1988).
93. C. B. Halsell, Organization of parabrachial nucleus efferents to the thalamus and amygdala in the golden hamster. *Journal of Comparative Neurology* **317**, 57–78 (1992).
94. H. Bester, J.-M. Besson, J.-F. Bernard, Organization of efferent projections from the parabrachial area to the hypothalamus: a Phaseolus vulgaris-leucoagglutinin study in the rat. *Journal of Comparative Neurology* **383**, 245–281 (1997).
95. M. Castle, E. Comoli, A. D. Loewy, Autonomic brainstem nuclei are linked to the hippocampus. *Neuroscience* **134**, 657–669 (2005).
96. H.-G. Jia, Z.-R. Rao, J.-W. Shi, An indirect projection from the nucleus of the solitary tract to the central nucleus of the amygdala via the parabrachial nucleus in the rat: a light and electron microscopic study. *Brain Research* **663**, 181–190 (1994).
97. J. M. Delfs, Y. Zhu, J. P. Druhan, G. S. Aston-Jones, Origin of noradrenergic afferents to the shell subregion of the nucleus accumbens: anterograde and retrograde tract-tracing studies in the rat. *Brain Research* **806**, 127–140 (1998).
98. J. Cadusseau, M. Roger, Afferent projections to the superior colliculus in the rat, with special attention to the deep layers. *J Hirnforsch* **26**, 667–681 (1985).
99. Y. Arima, S. Yokota, M. Fujitani, Lateral parabrachial neurons innervate orexin neurons projecting to brainstem arousal areas in the rat. *Sci Rep* **9**, 2830 (2019).
100. L. F. Kromer, R. Y. Moore, A study of the organization of the locus coeruleus projections to the lateral geniculate nuclei in the albino rat. *Neuroscience* **5**, 255–271 (1980).
101. J. M. Cedarbaum, G. K. Aghajanian, Afferent projections to the rat locus coeruleus as determined by a retrograde tracing technique. *Journal of Comparative Neurology* **178**, 1–15 (1978).
102. P.-H. Luppi, G. Aston-Jones, H. Akaoka, G. Chouvet, M. Jouvet, Afferent projections to the rat locus coeruleus demonstrated by retrograde and anterograde tracing with cholera-toxin B subunit and Phaseolus vulgaris leucoagglutinin. *Neuroscience* **65**, 119–160 (1995).

103. B. E. Jones, R. Y. Moore, Ascending projections of the locus coeruleus in the rat. II. Autoradiographic study. *Brain Research* **127**, 23–53 (1977).
104. M. Segal, V. Pickel, F. Bloom, The projections of the nucleus locus coeruleus: An autoradiographic study. *Life Sciences* **13**, 817–821 (1973).
105. S. G. Jeon, Y. J. Kim, K. A. Kim, I. Mook-Jung, M. Moon, Visualization of Altered Hippocampal Connectivity in an Animal Model of Alzheimer's Disease. *Mol Neurobiol* **55**, 7886–7899 (2018).
106. M.-A. Kim, H. S. Lee, B. Y. Lee, B. D. Waterhouse, Reciprocal connections between subdivisions of the dorsal raphe and the nuclear core of the locus coeruleus in the rat. *Brain Research* **1026**, 56–67 (2004).
107. A. Y. Deutch, M. Goldstein, R. H. Roth, Activation of the locus coeruleus induced by selective stimulation of the ventral tegmental area. *Brain Research* **363**, 307–314 (1986).
108. G. J. Ter Horst, P. de Boer, P. G. Luiten, J. D. van Willigen, Ascending projections from the solitary tract nucleus to the hypothalamus. A Phaseolus vulgaris lectin tracing study in the rat. *Neuroscience* **31**, 785–797 (1989).
109. S. Gasparini, J. M. Howland, A. J. Thatcher, J. C. Geerling, Central afferents to the nucleus of the solitary tract in rats and mice. *Journal of Comparative Neurology* **528**, 2708–2728 (2020).
110. A. J. Mcdonald, Organization of amygdaloid projections to the prefrontal cortex and associated striatum in the rat. *Neuroscience* **44**, 1–14 (1991).
111. A. L. Alhadeff, L. E. Rupprecht, M. R. Hayes, GLP-1 Neurons in the Nucleus of the Solitary Tract Project Directly to the Ventral Tegmental Area and Nucleus Accumbens to Control for Food Intake. *Endocrinology* **153**, 647–658 (2012).
112. R. M. Beckstead, J. R. Morse, R. Norgren, The nucleus of the solitary tract in the monkey: Projections to the thalamus and brain stem nuclei. *Journal of Comparative Neurology* **190**, 259–282 (1980).
113. H. Herbert, M. M. Moga, C. B. Saper, Connections of the parabrachial nucleus with the nucleus of the solitary tract and the medullary reticular formation in the rat. *Journal of Comparative Neurology* **293**, 540–580 (1990).
114. G. K. Aghajanian, R. Y. Wang, Habenular and other midbrain raphe afferents demonstrated by a modified retrograde tracing technique. *Brain Research* **122**, 229–242 (1977).
115. J. C. Geerling, A. D. Loewy, Aldosterone-sensitive neurons in the nucleus of the solitary tract: Efferent projections. *Journal of Comparative Neurology* **497**, 223–250 (2006).
116. L. Rinaman, Ascending projections from the caudal visceral nucleus of the solitary tract to brain regions involved in food intake and energy expenditure. *Brain Research* **1350**, 18–34 (2010).
117. E. J. Van Bockstaele, J. Peoples, P. Telegan, Efferent projections of the nucleus of the solitary tract to peri-locus coeruleus dendrites in rat brain: Evidence for a monosynaptic pathway. *Journal of Comparative Neurology* **412**, 410–428 (1999).
118. I. R. Kleckner, *et al.*, Evidence for a large-scale brain system supporting allostasis and interoception in humans. *Nature Human Behaviour* **1**, 1–14 (2017).
119. T. Caliński, J. Harabasz, A dendrite method for cluster analysis: Communications in Statistics: Vol 3, No 1. *Communications in Statistics* **3**, 1–27 (1974).
120. A. L. Cohen, *et al.*, Defining functional areas in individual human brains using resting functional connectivity MRI. *Neuroimage* **41**, 45–57 (2008).

121. V. Renvall, T. Witzel, L. L. Wald, J. R. Polimeni, Automatic cortical surface reconstruction of high-resolution T1 echo planar imaging data. *Neuroimage* **134**, 338–354 (2016).
122. J. R. Polimeni, *et al.*, Reducing sensitivity losses due to respiration and motion in accelerated echo planar imaging by reordering the autocalibration data acquisition. *Magnetic Resonance in Medicine* **75**, 665–679 (2016).
123. M. Bianciardi, *et al.*, In vivo functional connectome of human brainstem nuclei of the ascending arousal, autonomic, and motor systems by high spatial resolution 7-Tesla fMRI. *MAGMA* **29**, 451–462 (2016).
124. A. B. Satpute, *et al.*, Identification of discrete functional subregions of the human periaqueductal gray. *PNAS* **110**, 17101–17106 (2013).
125. F. Kurth, K. Zilles, P. T. Fox, A. R. Laird, S. B. Eickhoff, A link between the systems: functional differentiation and integration within the human insula revealed by meta-analysis. *Brain Struct Funct* **214**, 519–534 (2010).
126. P. J. Gianaros, L. K. Sheu, A review of neuroimaging studies of stressor-evoked blood pressure reactivity: emerging evidence for a brain-body pathway to coronary heart disease risk. *Neuroimage* **47**, 922–936 (2009).
127. R. M. Harper, *et al.*, fMRI responses to cold pressor challenges in control and obstructive sleep apnea subjects. *J Appl Physiol (1985)* **94**, 1583–1595 (2003).
128. T. D. Wager, *et al.*, Brain mediators of cardiovascular responses to social threat: part I: Reciprocal dorsal and ventral sub-regions of the medial prefrontal cortex and heart-rate reactivity. *Neuroimage* **47**, 821–835 (2009).
129. P. J. Gianaros, *et al.*, An inflammatory pathway links atherosclerotic cardiovascular disease risk to neural activity evoked by the cognitive regulation of emotion. *Biol Psychiatry* **75**, 738–745 (2014).
130. P. J. Gianaros, *et al.*, Individual differences in stressor-evoked blood pressure reactivity vary with activation, volume, and functional connectivity of the amygdala. *J Neurosci* **28**, 990–999 (2008).
131. K. Singh, *et al.*, Functional connectome of arousal and motor brainstem nuclei in living humans by 7 Tesla resting-state fMRI. *NeuroImage* **249**, 118865 (2022).
132. M. G. García-Gomar, *et al.*, In vivo Probabilistic Structural Atlas of the Inferior and Superior Colliculi, Medial and Lateral Geniculate Nuclei and Superior Olivary Complex in Humans Based on 7 Tesla MRI. *Frontiers in Neuroscience* **13** (2019).
133. K. Singh, *et al.*, Probabilistic Template of the Lateral Parabrachial Nucleus, Medial Parabrachial Nucleus, Vestibular Nuclei Complex, and Medullary Viscero-Sensory-Motor Nuclei Complex in Living Humans From 7 Tesla MRI. *Frontiers in Neuroscience* **13** (2020).
134. K. Singh, M. G. García-Gomar, M. Bianciardi, Probabilistic Atlas of the Mesencephalic Reticular Formation, Isthmic Reticular Formation, Microcellular Tegmental Nucleus, Ventral Tegmental Area Nucleus Complex, and Caudal–Rostral Linear Raphe Nucleus Complex in Living Humans from 7 Tesla Magnetic Resonance Imaging. *Brain Connectivity* **11**, 613–623 (2021).
135. D. Chen, *et al.*, The role of human superior colliculus in affective experiences during visual and somatosensory stimulation. [Preprint] (2022). Available at: https://www.biorxiv.org/content/10.1101/2022.12.09.519812v1 [Accessed 11 December 2022].

136. D. Chen, *et al.*, Layer-dependent activity in the human superior colliculus during working memory. [Preprint] (2022). Available at: https://www.biorxiv.org/content/10.1101/2022.12.06.518975v1 [Accessed 11 December 2022].
137. A. Morel, M. Magnin, D. Jeanmonod, Multiarchitectonic and stereotactic atlas of the human thalamus. *J Comp Neurol* **387**, 588–630 (1997).
138. J. E. Iglesias, *et al.*, A computational atlas of the hippocampal formation using ex vivo, ultra-high resolution MRI: Application to adaptive segmentation of in vivo MRI. *Neuroimage* **115**, 117–137 (2015).
139. W. M. Pauli, A. N. Nili, J. M. Tyszka, A high-resolution probabilistic in vivo atlas of human subcortical brain nuclei. *Sci Data* **5**, 180063 (2018).
140. B. Fischl, *et al.*, Whole Brain Segmentation. *Neuron* **33**, 341–355 (2002).
141. K. M. Lee, *et al.*, Functional Architecture of the Human Hypothalamus: Cortical Coupling and Subregional Organization Using 7-Tesla fMRI. *ArXiv* arXiv:2506.06191v1 (2025).
142. M. Bianciardi, *et al.*, Toward an In Vivo Neuroimaging Template of Human Brainstem Nuclei of the Ascending Arousal, Autonomic, and Motor Systems. *Brain Connect* **5**, 597–607 (2015).
143. G. Paxinos, H. Xu-Feng, G. Sengul, C. Watson, "Chapter 8 - Organization of Brainstem Nuclei" in *The Human Nervous System (Third Edition)*, J. K. Mai, G. Paxinos, Eds. (Academic Press, 2012), pp. 260–327.
144. T. Yarkoni, Big Correlations in Little Studies: Inflated fMRI Correlations Reflect Low Statistical Power—Commentary on Vul et al. (2009). *Perspect Psychol Sci* **4**, 294–298 (2009).
