# Supplementary material for: Cortical and subcortical mapping of the human allostatic–interoceptive system using 7 Tesla fMRI
Source: Nat Neurosci. 2025 Oct 23;28(11):2380–91. doi: 10.1038/s41593-025-02087-x (PMC12586188; doi:10.1038/s41593-025-02087-x)
Supplement: Supplementary file 2 — Reporting Summary [file 41593_2025_2087_MOESM2_ESM.pdf]

nature portfolio

Corresponding author(s): Jiahe Zhang

Last updated by author(s): Jul 16, 2025

Reporting Summary

Nature Portfolio wishes to improve the reproducibility of the work that we publish. This form provides structure for consistency and transparency in reporting. For further information on Nature Portfolio policies, see our Editorial Policies and the Editorial Policy Checklist.

Statistics

For all statistical analyses, confirm that the following items are present in the figure legend, table legend, main text, or Methods section.

n/a	Confirmed	
☐	☒	The exact sample size (n) for each experimental group/condition, given as a discrete number and unit of measurement
☐	☒	A statement on whether measurements were taken from distinct samples or whether the same sample was measured repeatedly
☐	☒	The statistical test(s) used AND whether they are one- or two-sided<br>*Only common tests should be described solely by name; describe more complex techniques in the Methods section.*
☐	☒	A description of all covariates tested
☐	☒	A description of any assumptions or corrections, such as tests of normality and adjustment for multiple comparisons
☐	☒	A full description of the statistical parameters including central tendency (e.g. means) or other basic estimates (e.g. regression coefficient) AND variation (e.g. standard deviation) or associated estimates of uncertainty (e.g. confidence intervals)
☐	☒	For null hypothesis testing, the test statistic (e.g. F, t, r) with confidence intervals, effect sizes, degrees of freedom and P value noted<br>*Give P values as exact values whenever suitable.*
☒	☐	For Bayesian analysis, information on the choice of priors and Markov chain Monte Carlo settings
☒	☐	For hierarchical and complex designs, identification of the appropriate level for tests and full reporting of outcomes
☐	☒	Estimates of effect sizes (e.g. Cohen's d, Pearson's r), indicating how they were calculated

Our web collection on statistics for biologists contains articles on many of the points above.

Software and code

Policy information about availability of computer code

Data collection	No software was used for data collection.
Data analysis	https://github.com/jiahez/7-Tesla-Allostatic-Interoceptive-System; Softwares used included Freesurfer v5.3.0 and dev version, FSL v5.0.7, AFNI 17.2.05, SPM8, MATLAB 2024b, ANTS, Brainstem Navigator v1.0, CANLAB Combined Atlas 2018

For manuscripts utilizing custom algorithms or software that are central to the research but not yet described in published literature, software must be made available to editors and reviewers. We strongly encourage code deposition in a community repository (e.g. GitHub). See the Nature Portfolio guidelines for submitting code & software for further information.

Data

Policy information about availability of data

All manuscripts must include a data availability statement. This statement should provide the following information, where applicable:

- Accession codes, unique identifiers, or web links for publicly available datasets
- A description of any restrictions on data availability
- For clinical datasets or third party data, please ensure that the statement adheres to our policy

https://openneuro.org/datasets/ds005747

Research involving human participants, their data, or biological material

Policy information about studies with human participants or human data. See also policy information about sex, gender (identity/presentation), and sexual orientation and race, ethnicity and racism.

Reporting on sex and gender	As this was a study of fundamental brain function across healthy individuals, we did not test the effects of sex or gender. We report information about participant's gender only to describe the characteristics of the sample. Participants reported gender (male, female) via self-report questionnaire.
Reporting on race, ethnicity, or other socially relevant groupings	As this was a study of fundamental brain function across healthy individuals, we did not test the effects of any socially constructed categorization variables. See above for discussion of gender.
Population characteristics	The sample consisted of native English-speaking adult participants (M age 26.9 ± 6.2 years old; 40 female, 50 male), with normal or corrected-to-normal vision and no history of neurological or psychiatric conditions.
Recruitment	Participants were recruited from the greater Boston area via electronic advertisements and paper flyers posted in local establishments. Because the 7 Tesla scanner used in the current study provides a more confined environment and higher field strength than standard 1.5 or 3 Tesla scanners, participants who chose to enroll and successfully completed the study may have had higher tolerance for those conditions. In addition, we enforced stricter safety screening (e.g., no permanent retainers and other implants that did not clear testing at 7 Tesla). The potential impact of these selection biases on the results is unclear.
Ethics oversight	All participants provided written informed consent and were compensated in accordance with the guidelines set by the institutional review board of Massachusetts General Hospital.

Note that full information on the approval of the study protocol must also be provided in the manuscript.

Field-specific reporting

Please select the one below that is the best fit for your research. If you are not sure, read the appropriate sections before making your selection.

☒ Life sciences ☐ Behavioural & social sciences ☐ Ecological, evolutionary & environmental sciences

For a reference copy of the document with all sections, see nature.com/documents/nr-reporting-summary-flat.pdf

Life sciences study design

All studies must disclose on these points even when the disclosure is negative.

Sample size	Target sample size was 160 for this project given power analysis for tasks not relevant to the current analyses. We stopped recrutiment at 140 due to equipment technical issues and COVID.
Data exclusions	Fifty participants were excluded from the current analysis (19 withdrew prior to the MRI session, three withdrew during the MRI acquisition due to discomfort, six did not complete scans due to online scan reconstruction failure, three did not complete scans due to time constraint, four were excluded due to other technical issues during acquisition, 10 were excluded due to scanner sequence error, four were excluded due to corrupted MRI data that could not be processed, and one was excluded due to excessive artifacts in the structural scan).
Replication	We randomly resampled 80% of the sample (N = 72) 1000 times to test for replication. Results from this bootstrapping analysis (thresholded at 950/1000) largely replicated the results observed with a single group analysis of the whole sample (N = 90).
Randomization	Participants were randomly assigned to one of two conditions for a task unrelated to current manuscript.
Blinding	Participants were randomized to complete other tasks that are not relevant to the current study.

Reporting for specific materials, systems and methods

We require information from authors about some types of materials, experimental systems and methods used in many studies. Here, indicate whether each material, system or method listed is relevant to your study. If you are not sure if a list item applies to your research, read the appropriate section before selecting a response.

Materials & experimental systems

n/a	Involved in the study
☒	☐ Antibodies
☒	☐ Eukaryotic cell lines
☒	☐ Palaeontology and archaeology
☒	☐ Animals and other organisms
☒	☐ Clinical data
☒	☐ Dual use research of concern
☒	☐ Plants

Methods

n/a	Involved in the study
☒	☐ ChIP-seq
☒	☐ Flow cytometry
☐	☒ MRI-based neuroimaging

Plants

Seed stocks

Report on the source of all seed stocks or other plant material used. If applicable, state the seed stock centre and catalogue number. If plant specimens were collected from the field, describe the collection location, date and sampling procedures.

Novel plant genotypes

Describe the methods by which all novel plant genotypes were produced. This includes those generated by transgenic approaches, gene editing, chemical/radiation-based mutagenesis and hybridization. For transgenic lines, describe the transformation method, the number of independent lines analyzed and the generation upon which experiments were performed. For gene-edited lines, describe the editor used, the endogenous sequence targeted for editing, the targeting guide RNA sequence (if applicable) and how the editor was applied.

Authentication

Describe any authentication procedures for each seed stock used or novel genotype generated. Describe any experiments used to assess the effect of a mutation and, where applicable, how potential secondary effects (e.g. second site T-DNA insertions, mosiacism, off-target gene editing) were examined.

Magnetic resonance imaging

Experimental design

Design type

Resting state

Design specifications

3 runs per session, each lasting 10 minutes.

Behavioral performance measures

No behavioral measures were collected during scans.

Acquisition

Imaging type(s)

functional, structural and diffusion

Field strength

7 Tesla

Sequence & imaging parameters

functional: fast low-angle excitation echo-planar technique, FOV=205mmx205mmx135.3mm, anterior to posterior, TE/TR/FA=28ms/2340ms/75
structural: multi-echo T1-weighted magnetization-prepared gradient-echo eco-planar image, FOV=205mmx206mm, TE/TR/FA=22ms/8520ms/90
diffusion: spin-echo echo-planar, FOV=205mmx205mmx67.1mm, transversal, TE/TR=63.2ms/5800ms

Area of acquisition

Whole brain (functional, structural), brainstem (diffusion)

Diffusion MRI ☒ Used ☐ Not used

Parameters

60 direction, b-values=1000s/mm2

Preprocessing

Preprocessing software

FSL version 5.0.7 (fslreorient2std, slicetimer, epi_reg, fslmeats, fslswapdim, fslroi, fslmaths) , AFNI version 17.2.05 (3dFourier), ANTs, SPM8, Freesurfer versions 5.3.0 (preproc-sess, mri_vol2surf), MATLAB 2024b

Normalization

Affine and non-linear transformation was computed using Advanced Normalization Tool (ANTs). The generic affine transformation was computed by concatenating center-of-mass alignment, rigid, similarity and fully affine transformations. The high-dimensional non-linear transformation was a symmetric diffeomorphic normalization transformation with neighorhood cross correlation, regulat sampling, gradient step size: 0.15, four multi-resolution levels, smoothing sigmas: 3,2,1,0 voxels - fixed image space, shrink factor: 6,4,2,1 voxels - fixed image space, histogram matching of images before registration, data winsorization - quantilte: .001, .999, convergnce criterion: slope of the normalized eneryg profile over the last 10 iterations < 10~-8. The affine and non-linear transformations were then combined into a single warp field and wer eapplied to the fMRI in t1wEPI space.

Normalization template	MNI305
Noise and artifact removal	6 motion parameters, white matter, 6 cerebrospinal fluid (lateral ventricle, inferior lateral ventricle, choroid plexus, third ventricle, fourth ventricle, cerebral aqueduct)
Volume censoring	We did not censor volumes.

Statistical modeling & inference

Model type and settings	mass univariate, fixed-effects at first and second levels
Effect(s) tested	One-sample t-test against zero in BOLD correlations

Specify type of analysis: ☐ Whole brain ☐ ROI-based ☒ Both

Anatomical location(s)	Cortical and amygdala ROIs were defined as 4mm-radius spheres centered on the MNI coordinates that showed increased activity in previous task-dependent fMRI studies of interoception. Brainstem ROIs were defined using the Brainstem Navigator probabilistic atlas. PAG ROIs were first manually defined in 20 individual subject based on their diffusion-weighted scans and then the group probabilistic map was thresholded at 35% to generate a group label.

Statistic type for inference (See Eklund et al. 2016)	voxel-wise
Correction	We relied on between-subsample replication and did not perform corrections between voxels.

Models & analysis

n/a	Involved in the study
☐	☒ Functional and/or effective connectivity
☒	☐ Graph analysis
☒	☐ Multivariate modeling or predictive analysis

Functional and/or effective connectivity	Pearson's correlation, Fisher's r-to-z transformed
